# CRISPR/Cas9-mediated deletion of Interleukin-30 suppresses IGF1 and CXCL5 and boosts SOCS3 reducing prostate cancer growth and mortality

**DOI:** 10.1186/s13045-022-01357-6

**Published:** 2022-10-13

**Authors:** Carlo Sorrentino, Luigi D’Antonio, Stefania Livia Ciummo, Cristiano Fieni, Lorena Landuzzi, Francesca Ruzzi, Simone Vespa, Paola Lanuti, Lavinia Vittoria Lotti, Pier Luigi Lollini, Emma Di Carlo

**Affiliations:** 1 Department of Medicine and Sciences of Aging, “G. d’Annunzio” University of Chieti-Pescara, Chieti, Italy; 2 Anatomic Pathology and Immuno-Oncology Unit, Center for Advanced Studies and Technology (CAST), “G. d’Annunzio” University of Chieti-Pescara, Via L. Polacchi 11, 66100 Chieti, Italy; 3 Laboratory of Experimental Oncology, IRCCS Istituto Ortopedico Rizzoli, Bologna, Italy; 4 Department of Experimental, Diagnostic and Specialty Medicine (DIMES), University of Bologna, Bologna, Italy; 5 Department of Experimental Medicine, “La Sapienza” University of Rome, Rome, Italy

**Keywords:** Prostate cancer, Interleukin-30, IGF1, CXCL5, CRISPR/Cas9, Tumor microenvironment

## Abstract

**Background:**

Metastatic prostate cancer (PC) is a leading cause of cancer death in men worldwide. Targeting of the culprits of disease progression is an unmet need. Interleukin (IL)-30 promotes PC onset and development, but whether it can be a suitable therapeutic target remains to be investigated. Here, we shed light on the relationship between IL30 and canonical PC driver genes and explored the anti-tumor potential of CRISPR/Cas9-mediated deletion of IL30.

**Methods:**

PC cell production of, and response to, IL30 was tested by flow cytometry, immunoelectron microscopy, invasion and migration assays and PCR arrays. Syngeneic and xenograft models were used to investigate the effects of IL30, and its deletion by CRISPR/Cas9 genome editing, on tumor growth. Bioinformatics of transcriptional data and immunopathology of PC samples were used to assess the translational value of the experimental findings.

**Results:**

Human membrane-bound IL30 promoted PC cell proliferation, invasion and migration in association with STAT1/STAT3 phosphorylation, similarly to its murine, but secreted, counterpart. Both human and murine IL30 regulated PC driver and immunity genes and shared the upregulation of oncogenes, BCL2 and NFKB1, immunoregulatory mediators, IL1A, TNF, TLR4, PTGS2, PD-L1, STAT3, and chemokine receptors, CCR2, CCR4, CXCR5. In human PC cells, IL30 improved the release of IGF1 and CXCL5, which mediated, via autocrine loops, its potent proliferative effect. Deletion of IL30 dramatically downregulated BCL2, NFKB1, STAT3, IGF1 and CXCL5, whereas tumor suppressors, primarily SOCS3, were upregulated. Syngeneic and xenograft PC models demonstrated IL30’s ability to boost cancer proliferation, vascularization and myeloid-derived cell infiltration, which were hindered, along with tumor growth and metastasis, by IL30 deletion, with improved host survival. RNA-Seq data from the PanCancer collection and immunohistochemistry of high-grade locally advanced PCs demonstrated an inverse association (chi-squared test, *p* = 0.0242) between IL30 and SOCS3 expression and a longer progression-free survival of patients with IL30^Neg^SOCS3^Pos^PC, when compared to patients with IL30^Pos^SOCS3^Neg^PC.

**Conclusions:**

Membrane-anchored IL30 expressed by human PC cells shares a tumor progression programs with its murine homolog and, via juxtacrine signals, steers a complex network of PC driver and immunity genes promoting prostate oncogenesis. The efficacy of CRISPR/Cas9-mediated targeting of IL30 in curbing PC progression paves the way for its clinical use.

**Supplementary Information:**

The online version contains supplementary material available at 10.1186/s13045-022-01357-6.

## Background

Prostate cancer (PC) is a major public health issue, which affects about 10 million men worldwide, and accounts for more than 400,000 deaths annually [1]. Aging of the population, due to the lengthening of the average life span, and reduced health care, due to the recent pandemic, will inevitably lead to an increase in PC incidence and mortality. Mortality is mainly due to metastatic disease for which, currently, there are no effective therapies. Identification of novel targetable drivers of PC onset and progression, to counteract metastasis and disease recurrences, is needed. Tumor onset and progression depend on the complex interactions between inherent germline susceptibility, acquired somatic gene alterations and microenvironmental factors [2], such as immunoregulatory mediators. Among them, Interleukin (IL)-30, which has been found to be expressed by cancer and/or infiltrating myeloid cells, in approximately 77% of metastatic PC, correlates with high-grade and stage of the disease [3] and has been reported to promote PC onset and progression in immunocompetent murine models [4]. Identified in 2002, as a partner of the Epstein-Barr virus induced gene 3 (EBI3), with which it forms the heterodimeric cytokine IL27 [5], IL30 can also behave as a self-standing cytokine, which is mainly produced by activated antigen-presenting cells and signals via IL6Rα (CD126), by recruiting a gp130 (CD130) homodimer [6].

Targeting of IL30, in both PC cells and host environment, consistently inhibited tumor growth, improved immune reactivity and prevented metastasis in mice [7]. In patients that underwent prostatectomy for locally advanced PC, those diagnosed with IL30^Negative^ tumor showed a favorable immune cell context, characterized by intra-tumoral TIA-1^+^CD4^+^T lymphocytes and rare Foxp3^+^Tregs, and a lower biochemical recurrence rate than patients bearing IL30^Positive^ tumor, in which IL30 was expressed in both tumor cells and infiltrating leukocytes [7].

Produced and released by murine PC stem-like cells (PC-SLC), IL30 has demonstrated to support their proliferation, self-renewal and tumorigenesis; however, its role in human prostate oncogenesis remains to be fully elucidated. To candidate IL30 as a target for personalized treatment of PC progression in patients with IL30 expressing tumor, we investigated its production by, and its effects on, human cancer cells derived from high-grade metastatic PC, by uncovering its potential to regulate PC driver genes, cancer immune escape mechanisms and its impact on host survival. Bioinformatic analyses of RNA-Seq data from the “Prostate Adenocarcinoma TCGA PanCancer” collection [8], corroborated by immunopathological studies of clinical PC samples, determined the prognostic impact of IL30 expression and its relationship with critical PC driver genes. Identification of IL30 as an upstream regulator of oncogenes, tumor suppressor and immunity genes, substantiates the use of CRISPR/Cas9-based deletion of IL30 to demolish the downstream tumor-promoting machinery and provides a new tool for the cure or prevention of advanced disease.

## Methods

### Cell cultures and MTT assay

Murine (m) prostatic intraepithelial neoplasia (PIN)-derived stem-like cells (PIN-SCs) were isolated from a 11-week-old TRAMP mouse [9] and characterized in refs. 10 and 11. PIN-SCs were authenticated by means of cell surface staining and flow cytometry for characteristic markers, and by their growth properties, as we described (4). Human PC cells, PC3 and DU145, and the murine PC cell line TRAMP-C1, isolated from a 32-week-old C57BL/6 male TRAMP mouse [12], were purchased from the American Type Culture Collection (ATCC, Manassas, VA, USA) and were authenticated by short tandem repeat profile analysis. All cell lines were passaged for fewer than 6 months after resuscitation and were confirmed mycoplasma-free by PCR analysis.

Cell lines were cultured in RPMI-1640 with 10% fetal calf serum (FCS; Seromed, Biochrom KG, Berlin, Germany). Cell viability and proliferation were assessed, using the CellTiter 96 AQueous One Solution Cell Proliferation Assay (#G3582; Promega, Madison, WI, USA), on TRAMP-C1 cells, after administration of recombinant murine IL30 (#7430-ML, R&D Systems, Minneapolis, MN, USA), and on PC3 and DU145 cells, after IL30 gene transfection or administration of recombinant human CXCL5 and IGF1 (#300-22 and #100-11, both from Peprotech, London, UK), or incubation with neutralizing anti-IL30, anti-CXCL5 (#AF1834, RRID:AB_355012 and #MAB254, RRID:AB_2261181, both from R&D Systems) and anti-IGF1 (#MAB2912, R&D Systems) antibodies (Abs). The anti-IGF1 Ab was provided by R&D Systems as a specific blocking antibody against human IGF1, as demonstrated by the neutralization curve reported at https://www.rndsystems.com/products/human-igf-i-igf-1-antibody-997121_mab2912 and included, as Figure S1, in Additional file 2.

### Migration and invasion assays

To evaluate the motility and the invasiveness of human and murine PC cells, CytoSelect Cell Migration and Invasion Assay (#CBA-100-C; Cell Biolabs, San Diego, CA, USA) was used according to manufacturer’s protocol.

### Flow cytometry

To assess phenotype markers, human and murine PC cells were harvested and mechanically dissociated into a single cell suspension. Then, the cells were pelleted, resuspended in PBS and incubated for 30 min, at 4 °C, with the Abs listed in the Supplemental Methods [see Additional file 1]. Acquisition was performed using a BD Scientific Canto II Flow Cytometer (RRID:SCR_018056), and the data were analyzed using FlowJo software (RRID:SCR_008520). Dead cells were excluded by 7AAD staining.

### Transfection with *IL27p28* (*IL30*) expressing vector

Creation of the IL30 expression lentiviral vector and its transfection into TRAMP-C1 cells were performed as described in the Supplemental Methods [see Additional file 1]. Expression of IL30 was confirmed by real-time RT-PCR, western blotting (WB) and ELISA assay.

For the overexpression of human IL30 in DU145 and PC3 cells, we used the IL27p28 Human Tagged ORF Clone (#RC209337L1; Origene, Rockville, MD, USA) which was transfected in cancer cells using Lipofectamine 3000 Reagent (#L3000001; Thermo Fisher Scientific, Waltham, MA, USA). The expression of IL30 was confirmed by real-time RT-PCR and WB.

### CRISPR/Cas9-mediated IL30 gene knockout

The CRISPR/Cas9 technology was used to generate IL30 knockout (IL30KO) murine PIN-SCs, and human DU145 and PC3 cells, as described in the Supplemental Methods [see Additional file 1]. IL30 gene knockout was validated by real-time RT-PCR, WB and ELISA assay (for murine PC cells).

### *STAT1* and *STAT3* knockdown experiments

For the silencing of *STAT1 and STAT3* genes in both DU145 and PC3 cell lines, we used the FlexiTube GeneSolution kit (#1027416, Qiagen, Hilden, Germany), according to the manufacturer’s instructions, and gene silencing efficiency was confirmed by WB. The AllStars siRNAs (Qiagen) were used as negative controls (scrambled siRNAs). Assessment of proliferation, migration and invasion abilities of IL30-overexpressing DU145 and PC3 cells, after STAT1 and STAT3 silencing, were performed as described in the Supplemental Methods [see Additional file 1].

### PCR array and real-time RT-PCR

PCR array and real-time RT-PCR were performed as described in the Supplemental Methods [see Additional file 1], using the RT^2^ Profiler Human Cancer Inflammation & Immunity Crosstalk PCR Array (#PAHS-181Z), the RT^2^ Profiler™ Human Prostate Cancer PCR Array (#PAHS-135Z), the RT^2^ Profiler Mouse Cancer Inflammation & Immunity Crosstalk PCR Array (#PAMM-181Z), the RT^2^ Profiler™ Mouse Prostate Cancer PCR Array (#PAMM-135Z), the Human_IL27p28_1_SG QuantiTect Primer Assay (#QT00236250) and the Mouse_Il27p28_1_SG QuantiTect Primer Assay (QT00143017) (all from Qiagen, Hilden, Germany).

### ELISA

Quantitation of IL30, CXCL5 and IGF1, in the supernatant derived from murine or human PC cells, was carried out using the following ELISA kits, according to manufacturer’s protocols: Human CXCL5/ENA-78 Quantikine ELISA Kit (#DX000, R&D Systems, Minneapolis, MN, USA); human IGF1 ELISA Kit (#ab211651, Abcam, Cambridge, UK); and mouse Interleukin-27 subunit alpha ELISA Kit (#CSB-E08466m, Cusabio, Wuhan, China).

### Western blotting

WB was performed to assess IL30 protein expression in mouse and human PC cells, and the regulation of phospho-STAT1, phospho-STAT3, STAT3, BCL2, NFKB1, DKK3 and SOCS3 in human PC cells, as described in the Supplemental Methods [see Additional file 1]. The protein bands were quantified using ImageJ software (RRID:SCR_003070).

### Mouse studies

C57BL/6J and NSG mice were purchased from Charles River (Wilmington, MA, USA). NSG mice were housed under high barrier conditions, according to the Jackson Laboratory’s guidelines, in the animal facility of the Center for Advanced Studies and Technology (CAST), at the "G. d'Annunzio" University of Chieti-Pescara, Italy. To evaluate the impact of IL30 overexpression on murine prostate cancer progression, three groups of thirty 8-week-old C57BL/6J mice were subcutaneously injected with 5 × 10^5^ wild-type (CTRL), Empty Vector (EV) or mIL30 lentiviral-DNA (IL30LV-DNA) transfected TRAMP-C1 cells. To study the effects of IL30 overexpression or knockout, with CRISPR/Cas9 technology, in an in vivo model of human prostate cancer, we subcutaneously injected three groups of thirty 8-week-old NSG mice with 3 × 10^5^ wild-type (CTRL), Empty Vector (EV) or hIL30 lentiviral-DNA (IL30LV-DNA) transfected DU145 cells, and another three groups of thirty 8-week-old NSG mice with 5 × 10^5^ wild-type (CTRL), non-targeting guide RNA-treated (NTgRNA) or IL30 knockout (IL30KO) DU145 cells. Tumors were measured with calipers as soon as they were palpable. Based on tumor growth and progression rate, 15 mice from each group were euthanized at key time points (3 mice per point) for histopathological analyses. The remaining 15 mice per group were kept until tumors reached 3 cm^3^ or evidence of suffering was observed. Autopsy and histopathological examinations of the different organs (liver, lungs, kidney and spleen) were performed. An overall sample size of 15 mice per group allowed the detection of a statistically significant difference in tumor growth, between three groups (ANOVA), with an 80% power, at a 0.05 significance level (G*Power, RRID:SCR_013726).

Animal procedures were performed in accordance with the European Community guidelines and were approved by the Institutional Animal Care Committee of “G. d’Annunzio” University and by the Italian Ministry of Health (Authorization n. 892/2018-PR).

### Bioinformatic analyses

For bioinformatic analyses, RNA sequencing (seq) data (obtained using the Illumina HiSeq 2000 RNA Sequencing System, Version 2) of tumor samples from the “Prostate Adenocarcinoma TCGA PanCancer” collection, which includes 494 PC cases (Table 1), were downloaded from the cBioPortal for Cancer Genomics database (https://www.cbioportal.org; cBioPortal, RRID:SCR_014555).

**Table 1:** Clinicopathological characteristics and expression profiles of IL30 and SOCS3 in prostate cancer patients included in the “Prostate Adenocarcinoma TCGA PanCancer collection”

Variables	*N*	IL30 expression		SOCS3 expression	
		High *n* (%)	Low *n* (%)	High *n* (%)	Low *n* (%)
*Age*					
41–50	35	1 (3)	34 (97)	32 (91)	3 (9)
51–60	187	4 (2)	183 (98)	186 (99)	1 (1)
61–70	235	10 (4)	225 (96)	225 (96)	10 (4)
71–80	37	1 (3)	36 (97)	34 (92)	3 (8)
Total	494	16 (3)	478 (97)	477 (97)	17 (3)
*Gleason score*					
6	43	0 (0)	43 (100)	41 (95)	2 (5)
7	242	5 (2)	237 (98)	236 (98)	6 (2)
8	68	4 (6)	64 (94)	64 (94)	4 (6)
9	136	7 (5)	129 (95)	131 (96)	5 (4)
10	5	0 (0)	5 (100)	5 (100)	0 (0)
Total	494	16 (3)	478 (97)	477 (97)	17 (3)
*Tumor size*					
pT2a	13	1 (8)	12 (92)	12 (92)	1 (8)
pT2b	10	1 (10)	9 (90)	10 (100)	0 (0)
pT2c	164	5 (3)	159 (97)	159 (97)	5 (3)
pT3a	157	5 (3)	152 (97)	150 (96)	7 (4)
pT3b	133	4 (3)	129 (97)	129 (97)	4 (3)
pT4	10	0 (0)	10 (100)	10 (100)	0 (0)
NA	7	0 (0)	7 (100)	7 (100)	0 (0)
Total	494	16 (3)	478 (97)	477 (97)	17 (3)
*Lymph node status*					
pN0	343	8 (2)	335 (98)	333 (97)	10 (3)
pN1	78	4 (5)	74 (95)	76 (97)	2 (3)
NA	73	4 (5)	69 (95)	68 (93)	5 (7)
Total	494	16 (3)	478 (97)	477 (97)	17 (3)
*Stage*					
I	10	1 (10)	9 (90)	9 (90)	1 (10)
II	130	4 (3)	126 (97)	129 (99)	1 (1)
III	199	3 (2)	196 (98)	191 (96)	8 (4)
IV	78	4 (5)	74 (95)	76 (97)	2 (3)
NA	77	4 (5)	73 (95)	72 (94)	5 (6)
Total	494	16 (3)	478 (97)	477 (97)	17 (3)

For each PC sample, the *Z*-score of the expression level for each gene of interest was calculated and compared to the mean of all the samples in the study. In the tumor samples from PC patients of the PanCancer collection, the expression of IL30 was never below a *Z*-score = − 2; therefore, samples with a *Z*-score ≥ 2 were defined as ***high-expressing***, whereas samples with a *Z*-score < 2 were defined as ***moderate-expressing***. In the same sample collection, the expression of SOCS3 was never higher than a *Z*-score = 2; therefore, samples with a *Z*-score ≤ − 2 were defined ***low-expressing***, whereas samples with a *Z*-score > − 2 were defined ***moderate-expressing*.**

Survival curves were constructed (with PC cases for which both gene expression and follow-up data were available) using the Kaplan–Meier method, and survival differences were analyzed by the Log-rank test. Gene co-occurrence analysis was performed by Fisher’s exact probability test, while Spearman’s correlation coefficient (*ρ*) was used to exclude correlations between gene expression and patient age, Gleason score and TNM staging.

All statistical tests were evaluated at an *α* level of 0.05, using Stata, version 13 (StataCorp, College Station, TX, USA; RRID:SCR_012763).

### Patients and samples

Tissue samples were collected and stored in the institutional biobank of the Local Health Authority n. 2 Lanciano Vasto Chieti, Italy, and the personal data processing complies with Data Protection Laws. For this study, prostate tissue samples were obtained from patients who underwent radical prostatectomy for PC, between 2009 and 2016, at the *Prostate Cancer Center* of the Local Health Authority. PC patients, ages 55–75, had not received immunosuppressive treatments, hormone- or radiotherapy, and were free from immune system diseases.

Clinic-pathological stages were determined according to the 8th edition of the TNM classification of malignant tumors (*Sobin LH, Gospodarowicz MK, Wittekind C. Wiley and Sons, Hoboken, NJ, USA 2017*), and tumor grade was assessed according to the Gleason scoring system from the prostate biopsies. Patients were followed-up for at least 5 years after prostatectomy, and biochemical recurrence (BCR) was defined as a PSA value > 0.2 ng/mL after prostatectomy, confirmed by another measurement after 4 weeks.

For this study, we examined 198 PC samples obtained from patients at Stage III (pT3N0M0, with negative surgical margins), and with a Gleason score of 8–10. After staining for IL30, we selected and then analyzed only the PC specimens that were found (i) to express IL30 in both PC cells and infiltrating leukocytes (referred to as IL30^Pos^PC; n. 52) or (ii) to lack IL30 expression in both PC cells and infiltrating leukocytes (referred to as IL30^Neg^PC; n. 123), according to the criteria that we defined previously [7] and described below. This sample size allowed the detection of a statistically significant difference between the two groups, with an 80% power and a 5% significance level.

The study was reviewed and approved by the Ethical Committee of the “G. d’Annunzio” University and Local Health Authority n.2 Lanciano Vasto Chieti, Italy (PROT. 1945/09 COET of 14/07/2009, amended in 2012). The study was performed, after written informed consent from patients, in accordance with the principles outlined in the Declaration of Helsinki.

### Histopathology, immunohistochemistry and morphometric analyses

For histology, tissue samples were fixed in 4% formalin, embedded in paraffin, sectioned at 4 μm and stained with hematoxylin and eosin (H&E).

Single or double immunostainings, on formalin-fixed and paraffin-embedded tissue sections, were performed as described in ref. 13, using the Abs listed in Table S1. Proliferation index, microvessel and cell counts were performed as described in the Supplemental Methods [see Additional file 1].

Expression of IL30 and SOCS3, in human PC specimens, was evaluated using the previously applied criteria [7], as described below.

*Expression of IL30 and SOCS3 by neoplastic cells* was evaluated using the following score, based on 1) the widening of the staining expressed as the percentage of tumor stained, i.e., < 50%, between 50 and 70%, and > 70%, and 2) the strength of the staining defined as absent (–), slight (±), distinct (+) or strong (++).

Thus, IL30 expression by neoplastic cells was defined as:

- *positive,* when (a) the widening was > 70% and its strength ranged from slight (±) to strong (++), or (b) the widening was between 50 and 70% and its strength ranged from distinct (+) to strong (++);
- *weakly positive,* when (a) the widening was between 50 and 70% and its strength was slight (±), or (b) the widening was equal to 50% and its strength ranged from slight (±) to strong (++);
- *negative* when the widening was < 50% and its strength was slight (±) to absent (–).

*Expression of IL30 and SOCS3 by infiltrating leukocytes* was evaluated using the following score, based on 1) the percentage of leukocyte expressing the cytokine, i.e., < 50%, between 50 and 70%, and > 70%, and 2) the strength of the cytokine staining that was defined as absent (–), scarce ( ±), distinct (+) or strong (++).

Thus, IL30 expression by infiltrating leukocytes was defined as:

- *strong,* when (a) the staining involved more than 70% of leukocytes and its strength ranged from scarce (±) to strong (++), or (b) the percentage of positively stained leukocytes was between 50 and 70% and the strength of the staining ranged from distinct (+) to strong (++);
- *distinct,* when (a) the staining involved > 50% and ≤ 70% of leukocytes and its strength was scarce (±), or (b) the staining involved 50% of leukocytes and its strength ranged from scarce (±) to strong (++);
- *scanty,* when the staining involved < 50% of leukocytes and its strength ranged from scarce (±) to absent (–).

Therefore, PC samples with *positive* and *strong* IL30, or SOCS3, expression were classified as IL30^Pos^, or SOCS3^Pos^, whereas PC samples with *negative* and *scanty* IL30, or SOCS3, expression were classified as IL30^Neg^, or SOCS3^Neg^.

Immunostained sections were examined by two pathologists in a blind fashion, with very good agreement (*κ* value = 0.89).

### Immunoelectron microscopy

PC cells were grown in monolayer and fixed in 2% PFA and 0.2% glutaraldehyde in 0.1 M PBS, pH 7.4, for 3 h at room temperature. Then, the cells were embedded into 12% gelatin in 0.1 M PBS, pH 7.4, solidified on ice, infused in 2.3 M sucrose overnight at 4 °C, mounted on aluminum pins and frozen in liquid nitrogen. Immunogold labeling was performed as described in the Supplemental Methods [see Additional file 1]. Labeled cryosections were analyzed with Philips CM10 and Fei-Philips Morgagni 268D transmission electron microscopes (Philips, Amsterdam, NL).

### Statistical analysis

For in vitro and in vivo studies, between-group differences were assessed by Student’s *t* test, or ANOVA, followed by Tukey HSD test.

For the bioinformatics, statistical analyses have been described above. Survival curves were constructed using the Kaplan–Meier method and survival differences were analyzed by the log-rank test. Gene co-occurrence analysis was performed by Fisher exact probability test. Spearman’s correlation coefficient (*ρ*) was used to exclude correlations between gene expression and patients’ age, Gleason score and TNM staging.

Follow-up time was 60 months. For morphometric studies, between-group differences were assessed by Student’s *t* test. All statistical tests were evaluated at an α level of 0.05 using Stata V.13 (StataCorp, College Station, TX, USA; RRID:SCR_012763).

## Results

### Human PC cells express membrane-bound Interleukin-30, which sustains their proliferation and invasiveness via STAT1/STAT3 signaling pathway

Assessment of IL30 production, throughout the natural history of PC, revealed that, in both mouse and human, it was confined to the rare PC-SLCs [4], in the early stages of the disease. In poorly differentiated, high-grade human PC and in metastatic lesions, IL30 was found in both cancer and infiltrating leukocytes [3]. While the consequences of targeting leukocyte-derived IL30 in the PC microenvironment have been thoroughly investigated [7, 14], the impact of cancer cell-derived IL30 in advanced human PC remains to be explored. To bridge this gap, two human (h) PC cell lines derived from metastases of high-grade PCs, DU145, endowed with a CK8/14^+^AR^+^PSA^+^ phenotype [15] (Additional file 2: Fig. S2A), and PC3, endowed with a CD44^+^AR^−^PSA^−^CgA^+^NSE^+^ neuroendocrine phenotype [16] (Additional file 2: Fig. S2B), were analyzed for their production of, and response to, IL30.

**Fig. 1:**
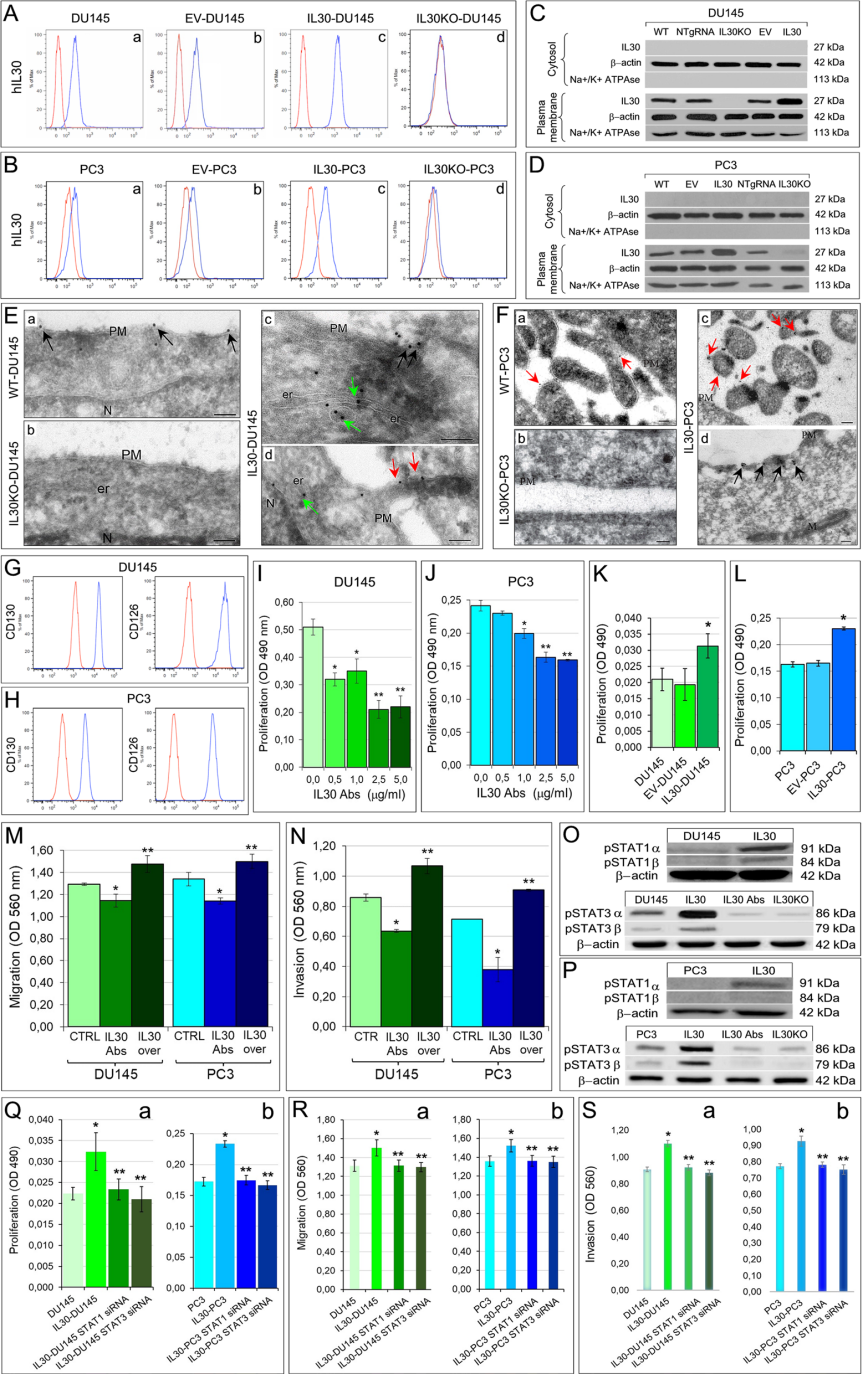
Constitutive expression of IL30 in human PC cells and IL30-dependent regulation of their proliferation, migration and invasion abilities. **A**, **B** Cytofluorimetric analyses of IL30 expression in human PC cells, DU145 (**A**) and PC3 (**B**). The DU145 cells showed a mean fluorescence intensity (MFI) ratio of 2.34, whereas the MFI ratio of PC3 cells was 1.95. The MFI was obtained calculating the ratio between the fluorescence of the samples and their isotype controls. Red lines: isotype control. Blue lines: anti-IL30 Abs. Results obtained from NTgRNA-treated cells were comparable with those from WT and EV-transfected cells. Experiments were performed in triplicate. **C**, **D** Western blot analyses of IL30 protein expression in the cytosolic and plasma membrane fractions of wild type, NTgRNA-treated, IL30KO, EV and IL30 gene-transfected DU145 (**C**) and PC3 (**D**) cells. **E**, **F** Cryo-immunoelectron microscopy of IL30 in DU145 (**E**) and PC3 (**F**) cells, showing IL30 localization, by gold particles, in WT (a), IL30KO (b) and IL30-overexpressing (c, d) cells. The gold particles were more frequent in IL30-overexpressing DU145 and PC3 cells (c, d) than in WT cells (a), whereas they were absent in IL30KO cells (b). In both DU145 and PC3 cells, the gold particles specifically delineated the plasma membranes (black arrows) and their microvilli-like structures (red arrows), the endoplasmic reticulum and associated cytoplasmic vesicles (green arrows). One out of four labeling experiments is shown. PM, plasma membrane; N, nucleus; er, endoplasmic reticulum; M, mitochondrion. Scale bars: 100 nm. **G**, **H** Cytofluorimetric analyses of gp130 (CD130) and IL6Rα (CD126) expression in DU145 (**G**) and PC3 (**H**) cells. Red lines: isotype control. Experiments were performed in triplicate. **I**, **J** MTT assay of DU145 (**I**) and PC3 (**J**) cells, after 48 h of treatment with anti-IL30 Abs (0.5–5.0 µg/mL). ANOVA: *p* < 0.0001. **I** *p < 0.01, Tukey HSD test compared with 0.0 µg/mL. **p < 0.05, Tukey HSD test compared with 0.0, 0.5 and 1.0 μg/mL. **J** *p < 0.01, Tukey HSD test compared with 0.0 and 0.5 µg/mL. **p < 0.01, Tukey HSD test compared with 0.0, 0.5 and 1.0 μg/mL. Results are expressed as mean ± SD. **K**, **L** MTT assay of IL30 gene-transfected, IL30-DU145 (K) and IL30-PC3 (L) cells, versus EV-transfected and WT cells. **K** ANOVA, *p* < 0.05. *p < 0.05, Tukey HSD test compared with WT and EV-transfected cells. **L** ANOVA, *p* < 0.0001. *p < 0.01, Tukey HSD test compared with WT and EV-transfected cells. Results are expressed as mean ± SD. **M**, **N** The treatment with anti-IL30 Abs (48 h) significantly decreased the number of DU145 and PC3 cells, that migrated (**M**) across the polycarbonate membrane insert or that invaded (**N**) the basement membrane matrix layer. By contrast, IL30 overexpression (IL30 over) significantly increased the number of migrating and invading DU145 (M) and PC3 (**M**) cells. Results obtained from EV-transfected cells are comparable with those from untreated wild-type cells (CTRL). Experiments were performed in triplicate. Results are expressed as mean ± SD. ANOVA, *p* < 0.01. *p < 0.05, Tukey HSD test compared with CTRL. **p < 0.05, Tukey HSD test compared with CTRL and cells treated with anti-IL30 Abs. **O**, **P** Quantitative western blot analysis of the expression of phospho-STAT1α and β isoforms, and phospho-STAT3α and β isoforms in DU145 (**O**) and PC3 (**P**) cells, and corresponding IL30 gene-transfected (IL30) cells, or IL30 gene knockout (IL30KO) cells, or wild-type cells treated with anti-IL30 Abs (IL30Abs). Expression of phospho-STAT1α was 15.93, and 53.26 times higher in IL30-DU145 and IL30-PC3, respectively, than in wild-type cells. Expression of phospho-STAT1β was 31.46, and 17.56 times higher in IL30-DU145 and IL30-PC3, respectively, than in wild-type cells. Expression of phospho-STAT3α and β was higher in IL30-DU145 (2.33 and 3.12 times) than in wild-type cells, whereas it was reduced in IL30KO-DU145 (− 6.63 and − 22.75 times) and in DU145 cells treated with anti-IL30 Abs (− 5.94 and − 23.89 times). Expression of phospho-STAT3α and β was higher in IL30-PC3 (2.22 and 4.17 times) than in wild-type cells, whereas it was reduced in IL30KO-PC3 (− 3.02 and − 6.42 times) and in PC3 cells treated with anti-IL30 Abs (− 2.96 and − 3.32 times). Results from control EV-transfected or NTgRNA-treated cells were comparable with those from wild-type cells. **Q** MTT assay of *STAT1* siRNA- or *STAT3* siRNA-transfected IL30-DU145 (a) and IL30-PC3 (b) cells. (a) ANOVA, *p* < 0.01; *p < 0.05, Tukey HSD test compared with DU145 cells; **p < 0.05, Tukey HSD test compared with IL30-DU145 cells. (b) ANOVA, *p* < 0.0001; *p < 0.01, Tukey HSD test compared with PC3 cells; **p < 0.01, Tukey HSD test compared with IL30-PC3 cells. Results from cells transfected with *STAT1* or *STAT3* scrambled siRNAs are comparable with those from IL30-overexpressing cells. Results are expressed as mean ± SD. **R** Migration assay of *STAT1* siRNA- or *STAT3* siRNA-transfected IL30-DU145 (a) and IL30-PC3 (b). ANOVA, *p* < 0.05. *p < 0.05, Tukey HSD test compared with DU145 (a) and PC3 (b) cells; **p < 0.05, Tukey HSD test compared with IL30-DU145 (a) and IL30-PC3 (b) cells. Results from cells transfected with *STAT1* or *STAT3* scrambled siRNAs are comparable with those from IL30-overexpressing cells. Results are expressed as mean ± SD. **S** Invasion assay of *STAT1* siRNA- or *STAT3* siRNA-transfected IL30-DU145 (a) and IL30-PC3 (b) cells. ANOVA, *p* < 0.0001. *p < 0.01, Tukey HSD test compared with DU145 (a) and PC3 (b) cells; **p < 0.01, Tukey HSD test compared with IL30-DU145 (a) and IL30-PC3 (b) cells. Results from cells transfected with *STAT1* or *STAT3* scrambled siRNAs are comparable with those from IL30-overexpressing cells. Results are expressed as mean ± SD

**Fig. 2:**
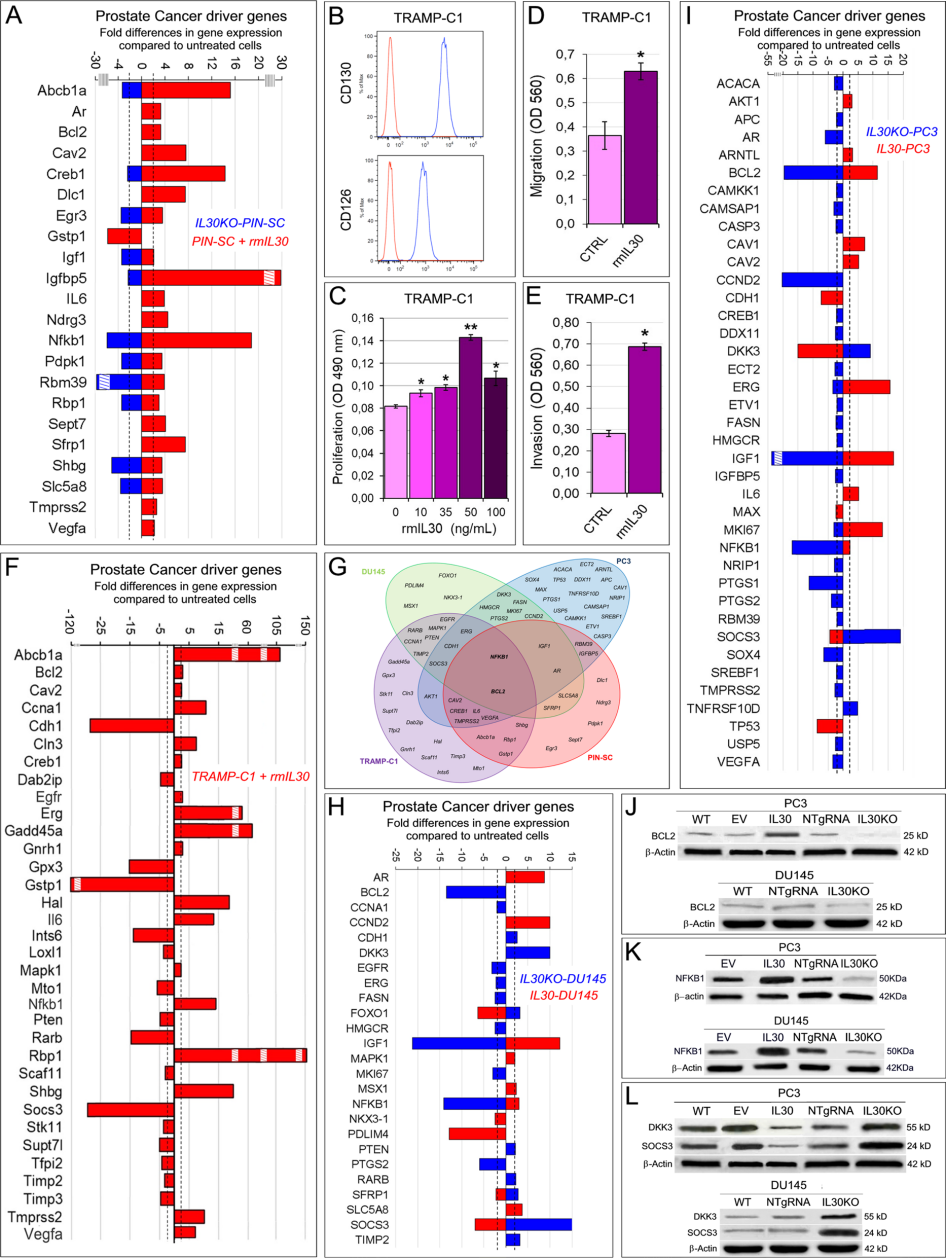
IL30-dependent regulation of PC driver genes in murine and human PC cells. **A** Mouse Prostate Cancer PCR Array. Fold differences of the mRNAs of PC driver genes between rmIL30-treated (red bars) and untreated wild-type murine (m) PC-SLCs, and between IL30KO-mPC-SLCs (blue bars) and mPC-SLCs. Results obtained from control NTgRNA-treated and untreated mPC-SLCs were comparable to those from wild type cells. A significant threshold of twofold change in gene expression corresponded to *p* < 0.001. Only genes with a fold change > 2, in at least one condition, up- or downregulation, are shown. Experiments were performed in duplicate. Stripped boxes represent scale breaks. The dashed lines represent the twofold change cutoff. **B** Cytofluorimetric analyses of gp130 (CD130) and IL6Rα (CD126) expression in TRAMP-C1 cells. Red lines: isotype control. Experiments were performed in triplicate. **C** MTT assay of TRAMP-C1 cells after 48 h treatment with rmIL30 at concentrations of 10, 35, 50 and 100 ng/mL. ANOVA: *p* < 0.0001. *p < 0.05, Tukey HSD test compared with 0 ng/mL. **p < 0.01, Tukey HSD test compared with 0, 10, 35 and 100 ng/mL. Results are expressed as mean ± SD. **D**, **E** The treatment with rmIL30 (6 h), significantly increased the number of TRAMP-C1 cells, which migrated (**D**) across the polycarbonate membrane insert, or which invaded (**E**) the basement membrane matrix layer. Results are expressed as mean ± SD. *Student’s *t* test: *p* = 0.0001 (**D**), *p* = 0.00001 (**E**), compared with untreated (CTRL) cells. Experiments were performed in triplicate. **F** Mouse Prostate Cancer PCR Array. Fold differences of the mRNAs of PC driver genes between rmIL30-treated (red bars) and untreated wild-type TRAMP-C1 cells. A significant threshold of a twofold change in gene expression corresponded to *p* < 0.001. Only genes with a fold change > 2 are shown. Experiments were performed in duplicate. Stripped boxes represent scale breaks. The dashed lines represent the twofold change cutoff. **G** Venn diagram representing the “PC Driver Genes” which are up- and/or downregulated by IL30 (treatment with rmIL30 or human IL30 gene knockout) in TRAMP-C1 (purple circle), PIN-SC (red circle), DU145 (green circle) and PC3 cells (blue circle). Overlapping circles illustrate the sharing of IL30-regulated genes between different cell lines. **H** Human Prostate Cancer PCR Array. Fold differences of mRNAs of PC driver genes between IL30-overexpressing IL30-DU145 cells (red bars) and wild-type DU145 cells, and between IL30KO-DU145 cells (blue bars) and wild-type cells. Results obtained from control NTgRNA-treated and EV-transfected DU145 cells were comparable to those from wild-type cells. A significant threshold of twofold change in gene expression corresponded to *p* < 0.001. Only genes with a fold change > 2, in at least one condition, up- or downregulation, are shown. Experiments were performed in duplicate. The dashed lines represent the twofold change cutoff. **I** Fold differences of the mRNAs of PC driver genes between IL30-overexpressing IL30-PC3 cells (red bars) and wild-type PC3 cells, and between IL30KO-PC3 cells (blue bars) and wild-type cells. Results obtained from control NTgRNA-treated and EV-transfected PC3 cells were comparable to those from wild-type cells. A significant threshold of a twofold change in gene expression corresponded to *p* < 0.001. Only genes with a fold change > 2, in at least one condition, up- or downregulation, are shown. Experiments were performed in duplicate. The stripped box represents a scale break. The dashed lines represent the twofold change cutoff. **J** Western blot analysis of BCL2 protein expression in WT, EV- or IL30 gene-transfected, NTgRNA-treated and IL30KO-PC3 cells, and in WT, NTgRNA-treated and IL30KO-DU145 cells. **K** Western blot analysis of NFKB1 protein expression in EV- or IL30 gene-transfected, NTgRNA-treated and IL30KO, PC3 and DU145 cells. Results obtained from control NTgRNA-treated and EV-transfected cells were comparable to those from wild-type cells. **L** Western blot analysis of DKK3 and SOCS3 protein expression in WT, EV- or IL30 gene-transfected, NTgRNA-treated and IL30KO-PC3 cells, and in WT, NTgRNA-treated and IL30KO-DU145 cells

Due to its tertiary structure [17], human IL30 (IL27p28 subunit, or IL27α) cannot be secreted [5], unless it heterodimerizes with soluble receptor-like proteins (EBI3 or Cytokine-Like Factor1) to form heterodimeric complexes [18]; therefore, we looked for its expression in the plasma membrane of hPC cells. Flow cytometry showed IL30 expression on the cell surface of both, PC3 and DU145, PC cells (Fig. 1A, B). These data were confirmed by WB analysis, which discriminated plasma membrane proteins from the cytoplasmic protein fraction (Fig. 1C, D), and by immunoelectron microscopy, which visualized IL30 expression in the endoplasmic reticulum-associated vesicles and on the plasma membranes, especially on the surface of microvilli-like structures (Fig. 1E, F). To assess whether membrane-bound IL30 can affect, via juxtacrine signaling, neighboring cancer cells, we analyzed their expression of gp130 and CD126, currently known to function as the IL30 receptor chains (8) (Fig. 1G, H), and determined their viability, both after adding neutralizing anti-IL30 Abs to the culture medium (Fig. 1I, J) and following IL30 overexpression (IL30-DU145 and IL30-PC3), obtained by gene transfection (Fig. 1K, L). A substantial inhibition, or increase, in cell proliferation (Fig. 1I, J, K, L), migration and invasion abilities (Fig. 1M, N), was detected in both hPC cell lines, upon abrogation or overexpression of IL30, respectively. In both cell lines, IL30 overexpression led to increased expression of the phosphorylated STAT1 and STAT3 isoforms (Fig. 1O, P), which were clearly suppressed in wild-type cells treated with anti-IL30 Abs, or in IL30KO cells. This finding suggests that, as observed for its soluble murine counterpart [5], the human membrane-bound form of IL30, expressed by either androgen dependent or independent PC cells, fosters tumor proliferation and invasiveness, through the activation of the STAT1/STAT3 signaling pathway [6, 19]. Silencing of STAT1 and STAT3 with specific siRNAs (Additional file 2: Fig. S2C, D) in IL30-overexpressing DU145 and PC3 cells (in which the increase of the phosphorylated isoforms of STAT1 and STAT3 was evident) resulted in a significant (ANOVA: *p* < 0.05) reduction in their proliferation, migration and invasion abilities, which were comparable to those of wild-type cells (Fig. 2Q, R, S), thus confirming, in human PC cells, the role of the STAT1/STAT3 pathway in IL30 signaling.

### Human membrane-bound and murine secreted Interleukin-30 regulates PC driver genes

Since neutralization of membrane-bound IL30, inhibits proliferation and invasiveness in human PC cells, while in the mouse model, targeting of the IL30 gene has demonstrated to inhibit murine PC-SLC tumorigenicity, tumor onset and progression [4], we wondered whether IL30 signaling might regulate, in both human and murine PC cells, the network of genes driving PC oncogenesis. We explored this possibility by performing PCR arrays for PC driver genes on both mouse and human PC cells, after treatment with recombinant IL30 (in murine cells) or knockout of the IL30 gene (in murine and human cells).

In PC-SLCs, isolated from PIN developed in a 11-week-old TRAMP mouse [11], hereinafter referred to as PIN-SCs, which express the IL30R and constitutively release IL30 [4], CRISPR/Cas9-mediated genomic deletion of *Il30*, that we have performed [see ref. 4], inhibited the expression of *Abcb1a* (− 3.30 times), *Creb1* (− 2.42 times), *Igf1* (− 3.45 times), *Igfbp5* (− 2.32 times), *Nfkb1* (− 5.97 times), *Pdpk1* (− 3.44 times), *Rbm39* (− 30.06 times), *Rbp1* (− 3.43 times) and *Shbg* (− 5.13 times), which were upregulated by the treatment with recombinant murine (rm) IL30. Treatment with IL30 also upregulated tumor progression genes, such as *Ar* (3.27 times), *Bcl2* (3.25 times), *IL6* (3.90 times), *Cav2* (7.60 times), *Ndrg3* (4.5 times), *Sept7* (4.10 times) and *Sfrp1* (7.46 times) (Fig. 2A).

In TRAMP-C1 cells, derived from a PC developed in a 32-week-old TRAMP mouse [12], which expressed CD126 and gp130 receptor chains (Fig. 2B), but did not release IL30, as assessed by ELISA, the treatment with rmIL30 (10–100 ng/mL) led to a significant increase of their proliferation (Fig. 2C. ANOVA: *p* < 0.0001; Tukey HSD test: *p* < 0.01), migration and invasion abilities (Fig. 2D, E. ANOVA: *p* < 0.001; Tukey HSD test: *p* < 0.01), as we previously observed in mPC-SLCs [4] and have shown here in hPC cells (Fig. 1K, L, M, N and ref. 3). The treatment of TRAMP-C1 cells with rmIL30 (50 ng/mL) substantially upregulated genes coding for *Abcb1a* (108.99 times), *Ccna1* (10.78 times), *Cln3* (7.35 times), *Erg* (56.41 times), *Gadd45a* (64.80 times), *Hal* (18.61 times), *IL6* (12.20 times), *Nfkb1* (14.60 times), *Rbp1* (151.17 times), *Shbg* (19.95 times), *Tmprss2* (10.13 times) *and Vegfa* (7.1 times), whereas the expression of a wide range of tumor suppressor genes, primarily, Cdh1 (− 28.65 times), *Gpx3* (− 15.34 times), *Gstp1* (− 120.5 times), *Ints6* (− 13.83 times), *Mto1* (− 5.82 times), *Pten* (− 4.86 times), *Rarb* (− 14.62 times) and *Socs3* (− 29.85 times), was suppressed (Fig. 2F).

In both murine PC cell lines, PIN-SCs and TRAMP-C1, isolated from the early and late stages, respectively, of prostate carcinogenesis of TRAMP mice, treatment with IL30 led to the upregulation of *Abcba1, Bcl2, Cav2, Creb1, IL6, Nfkb1, Rbp1, Shbg, Tmprss2 and Vegfa*, and the downregulation of *Gstp1* (Fig. 2G).

In human PC cells, DU145, abrogation of the constitutively produced IL30, by CRISPR/Cas9 genome editing (Fig. 1C), led to the upregulation of tumor suppressor genes, such as *CDH1 (*2.59 times), *DKK3* (10.03 times), *FOXO1* (3.21 times), *PTEN* (2.13 times), *RARB* (2.26 times), *SFRP1* (2.78 times), *TIMP2* (3.19 times) and especially *SOCS3* (15.20 times), whereas expression of oncogenes, such as *CCNA1, EGFR, ERG, FASN, HMGCR, MKI67, PTGS2* and, especially, *BCL2* (− 13.50 times), *NFKB1* (− 14.10 times) and *IGF1* (− 21.26 times) were suppressed (Fig. 2H). By contrast, IL30 overexpression (Fig. 1C), in DU145 cells, led to a significant upregulation of PC driver genes, such as *MAPK1, MSX1, SLC5A8* and, primarily, *AR* (8.71 times), *CCND2* (10.01 times) and *IGF1* (12.30 times), whereas *SFRP1, NKX3-1* and, especially*, FOXO1* (− 6.35 times), *PDLIM4* (− 12.90 times) and SOCS3 (− 7.02 times) were downregulated (Fig. 2H).

In human PC cells, PC3, IL30 gene deletion (Fig. 1D) reshaped a wide range of PC driver genes, among which *AR* (− 5.96 times), *CCND2* (− 20.25 times), *PTGS1* (− 11.31 times), *PTGS2* (− 3.88 times), *SOX4* (− 6.38 times), *VEGFA* (− 3.35 times) and especially *IGF1* (− 52.22 times) were inhibited, whereas a range of tumor suppressors were upregulated, such as TNFRSF10D (4.84 times), *DKK3* (9.03 times) and, primarily *SOCS3* (18.41 times).

IL30 overexpression, in PC3 cells (Fig. 1D), led to a significant upregulation of cancer driver genes, such as *AKT1, ARNTL, CAV1, CAV2, IL6,* and especially *ERG* (15.71 times), *MKI67* (13.12 times), *BCL2* (11.50 times) and *IGF1* (16.89 times), whereas the tumor suppressors, *MAX* (− 2.37 times), *CDH1* (− 7.24 times), *TP53* (− 8.61 times), DKK3 (− 15.10 times) and *SOCS3* (− 4.40 times) were downregulated (Fig. 2I).

**Fig. 3:**
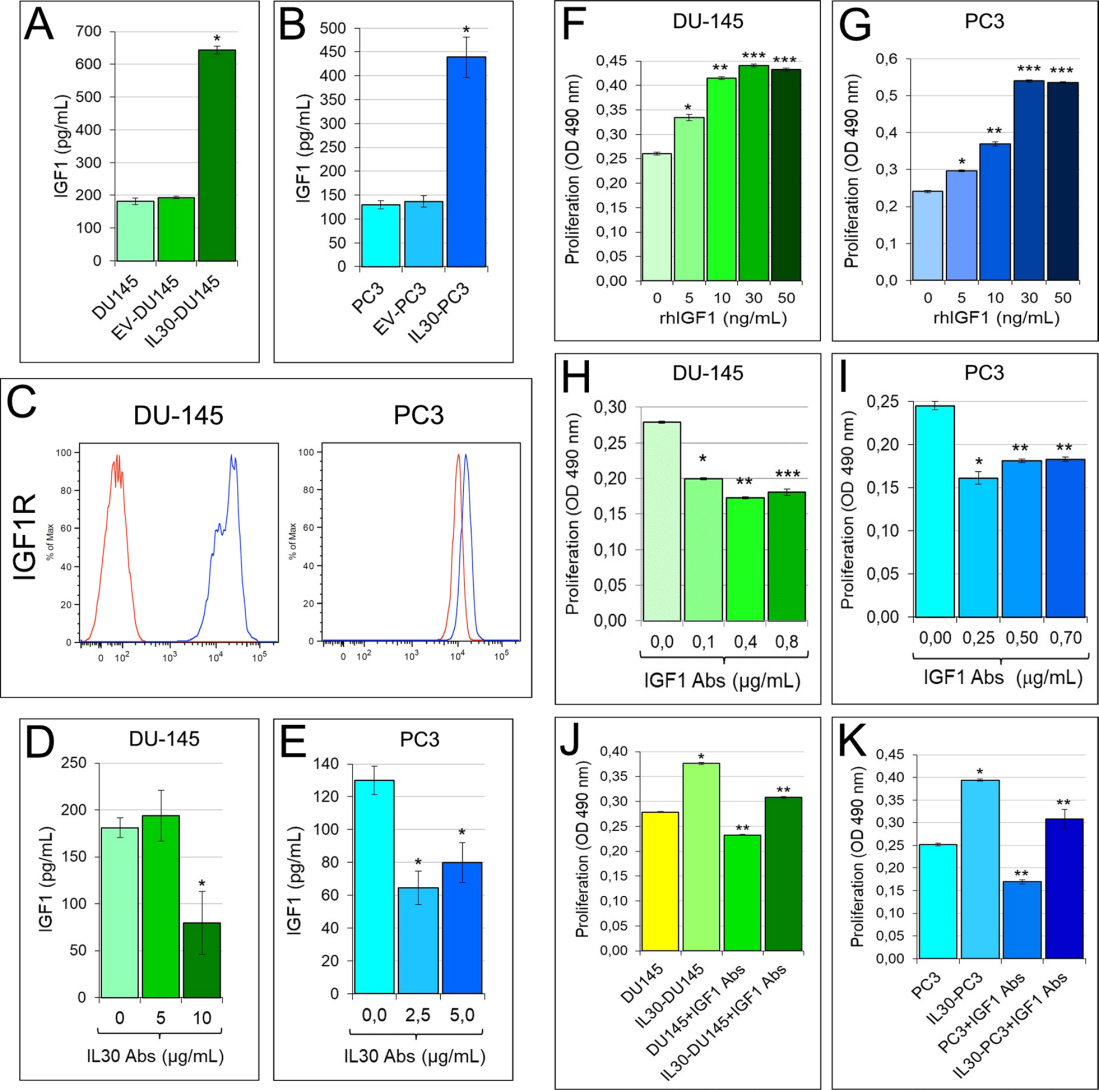
IL30-dependent regulation of IGF1 production in human PC cells and IGF1-mediated autocrine growth loop. **A**, **B** Elisa assay of IGF1 release by wild-type, EV and IL30 gene-transfected DU145 (**A**) and PC3 (**B**) cells**.** ANOVA: *p* < 0.0001. *p < 0.01, Tukey HSD test compared with WT and EV-transfected cells. Results are expressed as mean ± SD. **C** Cytofluorimetric analyses of IGF1R expression in PC3 and DU145 cells. Red lines: isotype control. Experiments were performed in triplicate. **D**, **E** Elisa assay of IGF1 release by wild-type DU145 (**D**) and PC3 (**E**) cells, after the treatment with anti-IL30 Abs. (D) ANOVA: *p* < 0.05. *p < 0.05, Tukey HSD test compared with DU145 cells untreated or treated with 5 μg/mL. **E** ANOVA: *p* < 0.001. *p < 0.01, Tukey HSD test compared with untreated PC3 cells. Results are expressed as mean ± SD. **F**, **G** MTT assay of DU145 (**F**) and PC3 (**G**) cells, untreated (0.0 ng/mL) or treated with rhIGF1 (5.0, 10, 30, 50 ng/mL). ANOVA: *p* < 0.0001. *p < 0.01, Tukey HSD test compared with 0 ng/mL. **p < 0.01, Tukey HSD test compared with 0 and 5 ng/mL. ***p < 0.01, Tukey HSD test compared with 0, 5 and 10 ng/mL. Results are expressed as mean ± SD. **H**, **I** MTT assay of DU145 (H) and PC3 (I) cells, untreated (0.0 μg/mL) or treated with anti-IGF1 Abs (0.1, 0.4, 0.8 μg/mL in DU145; 0.25, 0.50, 0.70 μg/mL in PC3). (H) ANOVA: *p* < 0.0001. *p < 0.01, Tukey HSD test compared with 0.0 μg/mL. **p < 0.01, Tukey HSD test compared with 0.0 and 0.1 μg/mL. ***p < 0.05, Tukey HSD test compared with 0.0, 0.1 and 0.4 μg/mL. (I) ANOVA: *p* < 0.0001. *p < 0.01, Tukey HSD test compared with 0.00 µg/mL. **p < 0.01, Tukey HSD test compared with 0.00 and 0.25 μg/mL. Results are expressed as mean ± SD. **J**, **K** MTT assay of wild-type and IL30 gene-transfected DU145 (**J**) and PC3 (**K**) cells, untreated (0.0 μg/mL), or treated with anti-IGF1 Abs (30 μg/mL). ANOVA: *p* < 0.0001. *p < 0.01, Tukey HSD test compared with wild-type cells. **p < 0.01, Tukey HSD test compared with wild-type and IL30-transfected cells. Results are expressed as mean ± SD

Noteworthy, both human PC cell lines, in which the IL30 gene was knocked out, shared (Fig. 2G) the downregulation of *ERG, MKI67, FASN, HMGCR, PTGS2* and a consistent suppression of *BCL2* (− 19.62 times in PC3 and − 13.50 in DU145) (Fig. 2J), *NFKB1* (− 16.40 times in PC3 and − 14.10 times in DU145) (Fig. 2K) and *IGF1* expression (52.22 times in PC3 and − 21.26 times in DU145), whereas *DKK3* (9.03 times in PC3 and 10.03 times in DU145) and, primarily, *SOCS3* (18.41 times in PC3 and 14.20 times in DU145) were upregulated (Fig. 2L). Furthermore, IL30-overexpressing DU145 and PC3 cells shared a significant upregulation of NFKB1 and IGF1 expression, confirmed at the protein level by WB (Fig. 2K) and ELISA assay, (Fig. 3A, B), respectively.

### IGF1 autocrine loop contributes to the IL30 driven proliferation of human PC cells

IGF1 is involved not only in the growth and development of the prostate gland, but also in the growth and progression of PC [20, 21]. High serum levels of IGF1 have been associated with an increased risk for PC [22, 23].

We found that both human PC cells, DU145 and PC3, constitutively produced and released IGF1, 181.11 ± 10.18 pg/mL, and 130.00 ± 8.82 pg/mL, respectively, and that they also expressed IGF1R (Fig. 3C). Both PC cell lines showed a reduced secretion of IGF1, after the blockade, with neutralizing anti-IL30 Abs, of their constitutive production of IL30 (DU145: 79.44 ± 33.39 vs 181.11 ± 10.18 pg/mL; PC3: 64.44 ± 10.18 vs 130.0 ± 8.82 pg/mL. ANOVA: *p* < 0.01; Tukey HSD test: *p* < 0.05. Fig. 3D, E). By contrast, the release of IGF1 increased, in both PC cell lines engineered to overexpress IL30, compared to control cells (IL30-DU145: 643.33 ± 12.02 pg/mL vs EV-DU145: 193.33 ± 3.33 pg/mL, and DU145 cells: 181.11 ± 10.18 pg/mL. ANOVA: *p* < 0.0001; Tukey HSD test: *p* < 0.01 vs both EV and WT. IL30-PC3: 438.89 ± 42.21 pg/mL vs EV-PC3: 136.67 ± 12.02 pg/mL, and PC3 cells: 130.00 ± 8.82 pg/mL. ANOVA: *p* < 0.0001; Tukey HSD test: *p* < 0.01 vs both EV and WT. Fig. 3A, B). The treatment of both, DU145 and PC3 cells, with recombinant human (rh) IGF1 increased, in a dose-dependent manner, their proliferation (ANOVA: *p* < 0.0001; Tukey HSD test: *p* < 0.01. Fig. 3F, G), which was suppressed by the treatment with anti-IGF1 Abs (ANOVA: *p* < 0.0001; Tukey HSD test: *p* < 0.01. Fig. 3H, I).

The increased proliferation of DU145 and PC3 cells induced by IL30 overproduction, obtained by IL30 gene transfection, was suppressed by anti-IGF1 Abs, which also inhibited the spontaneous proliferative activity of wild-type and control cells (Fig. 3J, K). Therefore, IGF1 acts as an autocrine growth factor for both AR^+^ and AR^−^ PC cells and accounts for much of the proliferative effect due to IL30.

### CRISPR/Cas9-targeted deletion of human Interleukin-30 upregulates SOCS3 and inhibits tumor production of IGF1 and PC progression improving survival of tumor-bearing host

To evaluate, in vivo*,* the consequences on PC development of IL30 expression, or targeted deletion, in human PC cells, DU145 cells, which constitutively expressed the highest level of IL30 (Fig. 1A, a), were implanted in NSG (NOD scid gamma) mice, after targeted deletion, or overexpression, of IL30 gene.

**Fig. 4:**
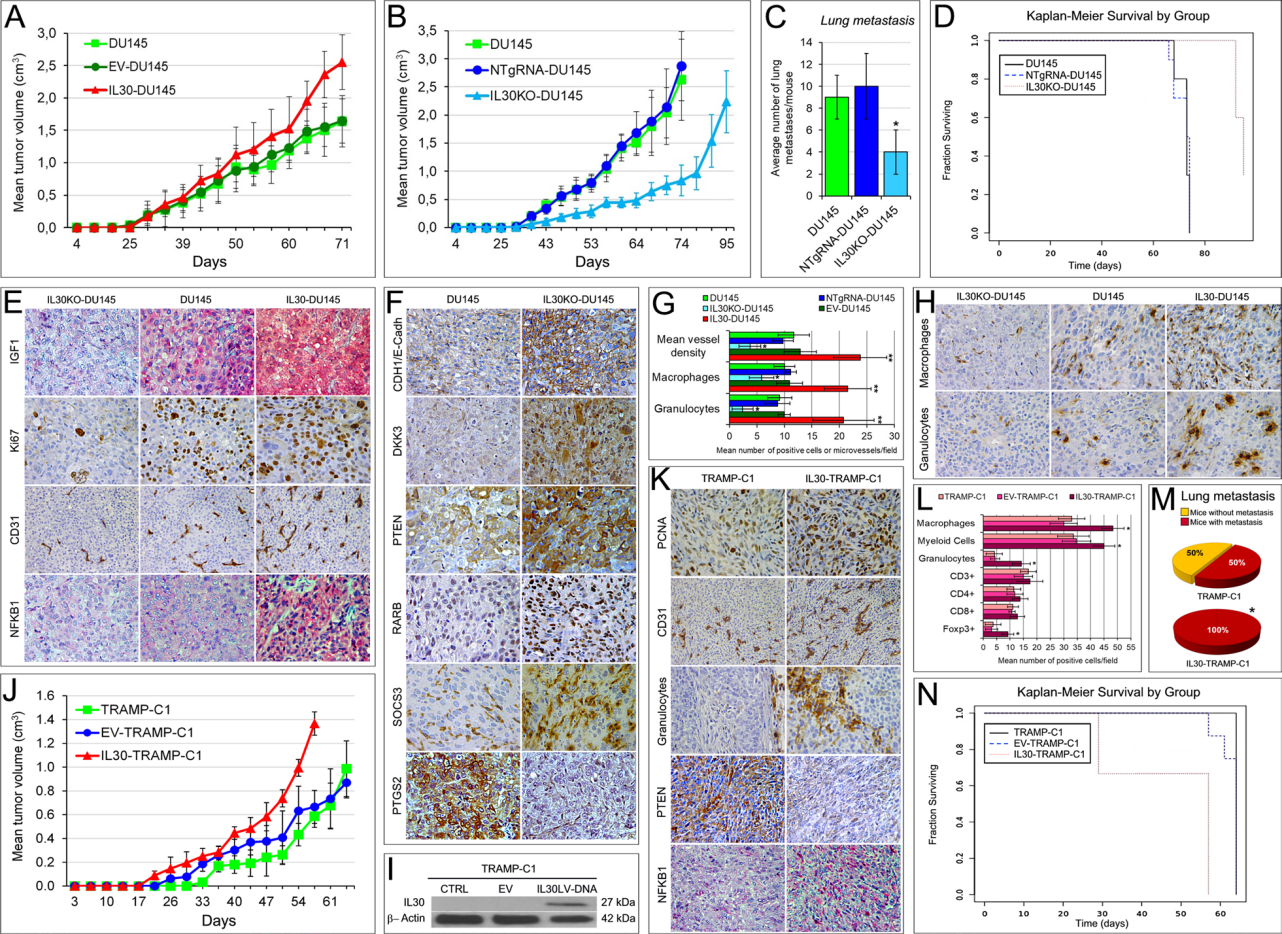
Tumor growth and survival of mice-bearing IL30-deficient or IL30-overexpressing PC of murine or human origin. **A** Mean volume of tumors developed in NSG mice, after s.c. implantation of wild type, EV- or IL30-DU145 cells. ANOVA, *p* < 0.0001; Tukey HSD test, *p* < 0.01 versus wild type or EV-transfected DU145 cells. Results are expressed as mean ± SD. **B** Mean volume of tumors developed in NSG mice, after s.c. implantation of wild type, NTgRNA-treated or IL30KO-DU145 cells. ANOVA, *p* < 0.0001; Tukey HSD test, *p* < 0.01 versus wild type or NTgRNA-treated DU145 cells. Results are expressed as mean ± SD. **C** Average number of lung metastasis spontaneously developed in NSG mice, which developed tumors after s.c. implantation of wild type, NTgRNA-treated or IL30KO-DU145 cells. ANOVA: *p* < 0.0001. *p < 0.01, Tukey HSD test versus DU145 or NTgRNA-treated DU145 cells. Results are expressed as mean ± SD. **D** Kaplan–Meier survival curves of mice-bearing tumors developed after s.c. implantation of wild type, NTgRNA-treated or IL30KO-DU145 cells. Log-rank test: *p* = 0.000009. **E** Immunopathological features of tumors developed in NSG mice, after s.c. implantation of IL30KO-DU145, wild type DU145 and IL30-DU145 cells. Expression of IGF1, proliferation (Ki67 Abs) and vascularization (CD31 Abs) were prominent in IL30-overexpressing tumors, and scanty in IL30KO tumors, compared with wild type tumors. Cytoplasmic and nuclear expression of NFKB1 was strong in IL30-overexpressing tumors and faint in IL30KO tumors. The immunopathological features of tumors developed after implantation of control, EV-transfected or NTgRNA-treated, cells were comparable to those of wild type tumors. Magnification: × 400; CD31, × 200. **F** Immunopathological features of tumors developed in NSG mice, after s.c. implantation of IL30KO-DU145 and wild type DU145 cells. Expression of tumor suppressor genes CDH1/E-Cadh, DKK3, PTEN, RARb and SOCS3 was stronger in IL30KO-DU145 tumors, when compared to wild type tumors, whereas the expression of PTGS2 was weaker. The immunopathological features of control tumors, developed in NSG after implantation of NTgRNA-treated cells, were comparable to those of wild type tumors. Magnification: × 400. **G** Automated immune cell count and microvessel density in tumors developed in NSG mice, after implantation of wild type, EV- or IL30 gene-transfected, NTgRNA-treated and IL30KO-DU145 cells, assessed by immunohistochemistry, as described in Methods. ANOVA, *p* < 0.0001. *p < 0.01, Tukey HSD test compared with DU145, NTgRNA-treated DU145 and EV-transfected DU145. **p < 0.01, Tukey HSD test compared with DU145, NTgRNA-treated DU145, IL30KO-DU145 and EV-transfected DU145. Results are expressed as mean ± SD. **H** The immune cell contexture of tumors developed in NSG mice, after s.c. implantation of IL30KO and IL30-DU145 cells, revealed a higher content of macrophages (anti-F4/80 Abs) and granulocytes (anti-Ly-6G Abs) in IL30-overexpressing tumors, compared to wild type tumors. By contrast, these immune cell populations were scarce to absent in IL30-deficient tumors. Magnification: × 400. Scale bars: 30 μm. **I** Western blot analysis of IL30 protein expression in wild type, EV- and IL30 gene-transfected TRAMP-C1 cells. **J** Mean volume of tumors developed in C57BL/6J mice, after s.c. implantation of wild type, EV- or IL30-TRAMP-C1 cells. ANOVA, *p* < 0.0001. Tukey HSD test, *p* < 0.01 versus wild type or EV-TRAMP-C1 cells. Results are expressed as mean ± SD. **K** Immunopathological features of tumors developed in C57BL/6J mice, after s.c. implantation of wild type or IL30-TRAMP-C1 cells revealed that proliferation (PCNA Abs), microvascular density (CD31 Abs) and granulocyte content (Ly-6G Abs) were higher in IL30-overexpressing tumors, than in control tumors. Cancer cells that formed IL30-TRAMP-C1 tumors showed reduced cytoplasmic expression of PTEN and an increased nuclear and cytoplasmic expression of NFKB1. Magnification: × 400; CD31, × 200. **L** Automated immune cell count in tumors developed in C57BL/6J mice, after s.c. implantation of wild type, EV or IL30 gene-transfected TRAMP-C1 cells, assessed by immunohistochemistry, as described in Methods. ANOVA, *p* < 0.001. *p < 0.01, Tukey HSD test compared with wild type or EV-TRAMP-C1 cells. Results are expressed as mean ± SD. **M** Percentage of lung metastasis spontaneously developed in C57BL/6J mice, which developed tumors after s.c. implantation of wild type or IL30 gene-transfected TRAMP-C1 cells. *Fisher’s exact test, *p* = 0.03 versus EV-TRAMP-C1 or TRAMP-C1. Results from mice implanted with EV-TRAMP-C1 cells were comparable to those obtained from mice implanted with wild type cells. **N** Kaplan–Meier survival curves of mice-bearing tumors developed after s.c. implantation of wild type, EV- or IL30 gene-transfected TRAMP-C1 cells. Log-rank test: *p* = 0.000124

The slow growing tumors developed after the subcutaneous (s.c.) implantation of wild type, or EV-DU145 cells, reached, 71 days later, a mean tumor volume (MTV) that was smaller than that of IL30-overexpressing DU145 tumors (1.639 ± 0.397 cm^3^ and 1.646 ± 0.340 cm^3^ vs 2.552 ± 0.421 cm^3^; ANOVA, *p* < 0.0001; Tukey HSD test, *p* < 0.01 vs both controls. Fig. 4A), but significantly bigger than that of IL30KO tumors (2.628 ± 0.718 cm^3^ and 2.873 ± 0.615 cm^3^ vs 0.837 ± 0.275 cm^3^; ANOVA, *p* < 0.0001; Tukey HSD test, *p* < 0.01 vs both controls. Fig. 4B).

Although all tumor-bearing mice developed lung metastases, the average number of metastases per mouse was significantly lower in mice-bearing IL30KO-DU145 tumors than in mice-bearing control NTgRNA-treated DU145 or wild-type DU145 tumors (4 vs 10 and 9, respectively) (ANOVA, *p* < 0.0001; Tukey HSD test, *p* < 0.01 vs both controls. Fig. 4C) and did not correlate with the tumor volume (Pearson correlation coefficient: *r* = 0.18). Moreover, Kaplan–Meier analysis revealed that mice-bearing IL30KO-DU145 tumors survived longer (95 days) than mice-bearing NTgRNA-treated DU145 or DU145 tumors (both groups, 74 days. Log-rank test: *p* < 0.0001. Fig. 4D).

Consistent with the transcriptional profile of PC driver genes in hPC cells, which overexpressed or lacked IL30, the production of IGF1 was considerable in IL30-overexpressing DU145 tumors, whereas it was scanty to absent in IL30-deficient DU145 tumors (Fig. 4E). IL30-overexpressing DU145 tumors also showed higher proliferation (Ki67: 66.03% ± 7.98% vs 48.43%% ± 7.14% and 49.27% ± 4.86%; ANOVA, *p* < 0.0001; Tukey HSD test, *p* < 0.01 vs both controls) and microvessel density (MVD: 23.79 ± 4.85 vs 11.71 ± 2.87 and 12.87 ± 2.94; ANOVA, *p* < 0.0001; Tukey HSD test, *p* < 0.01 vs both controls) than control tumors. By contrast, IL30-deficient tumors were low proliferating (Ki67: 25.00% ± 8.93% vs 48.43% ± 7.14% and 47.14% ± 6.69%; ANOVA, *p* < 0.0001; Tukey HSD test, *p* < 0.01 vs both controls), and poorly vascularized (MVD: 3.75 ± 1.98 vs 11.71 ± 2.87 and 9.75 ± 1.91; ANOVA, *p* < 0.0001; Tukey HSD test, *p* < 0.01 vs both controls. Fig. 4E).

Immunopathological analyses revealed that cytoplasmic and nuclear expression of NFKB1 was relevant in IL30-overexpressing tumors and scanty in IL30KO tumors, in which the expression of tumor suppressors CDH1/E-Cadherin, DKK3, PTEN, RARB and SOCS3, which was also detected in macrophage-like cells, was stronger than in control tumors, while that of PTGS2 was fainter (Fig. 4F), therefore, strengthening the in vitro data on the regulation of PC driver genes by IL30 in human PC cells.

PC cell production of IL30 also affected the extent of the intra-tumoral immune cell infiltrate, since macrophages and granulocytes increased in IL30-overexpressing tumor xenograft, compared to controls (ANOVA, *p* < 0.0001*;* Tukey HSD test, *p* < 0.01 vs wt-DU145 and EV-DU145), whereas they were poorly detected in IL30-deficient tumors (*ANOVA, p* < 0.0001*;* Tukey HSD test, *p* < 0.01 vs wt-DU145 and NTgRNA-treated DU145 (Fig. 4G, H).

To overcome the lack of specific immunity cells (T, B and NK) in the microenvironment of the tumor xenograft, and the failure of this model in assessing the role of such immune cells in IL30-conditioned tumor, we then used a fully immunocompetent model of cancer induced by TRAMP-C1 cells in C57BL/6J host. TRAMP-C1 cells, which lack constitutive IL30 secretion, were engineered to overexpress IL30, and the clone releasing 458 ± 12,06 ng/mL of mIL30, hereinafter referred to as IL30-TRAMP-C1 (Fig. 4I), was used for in vivo studies. After their s.c. implantation, in syngeneic host, IL30-TRAMP-C1 cells gave rise to tumors that grew faster than controls, and that reached, 57 days later, a MTV that was significantly higher (1.366 ± 0.098 cm^3^ vs 0.587 ± 0.094 cm^3^ and 0.665 ± 0.139 cm^3^; ANOVA, *p* < 0.0001; Tukey HSD test, *p* < 0.01 vs both controls (Fig. 4J). IL30-overexpressing TRAMP-C1 tumors were highly proliferating (PCNA: 65.17% ± 5.00% in IL30-TRAMP-C1 tumors vs 28.14% ± 4.78% in TRAMP-C1, and 27.71% ± 4.07% in EV-TRAMP-C1; ANOVA, *p* < 0.0001; Tukey HSD test, *p* < 0.01 vs both controls) and well vascularized (MVD: 20.71 ± 3.09 in IL30-TRAMP-C1 tumors vs 14.43 ± 2.72 in TRAMP-C1, and 13.57 ± 1.27 in EV-TRAMP-C1; ANOVA, *p* < 0.001; Tukey HSD test, *p* < 0.01 vs both controls. Fig. 4K). In accordance with the upregulation of NFKB1 detected in IL30 treated TRAMP-C1 cells in vitro, IL30-overexpressing TRAMP-C1 tumors revealed high level of expression of NFKB1 and a significant infiltrate of F4/80^+^ macrophages, CD11b^+^Gr-1^+^ myeloid-derived cells (MDC), Ly-6G^+^ granulocytes and Foxp3^+^ Tregs (Fig. 4K, L), whereas infiltration of CD3^+^T, as well as that of CD8^+^ and CD4^+^ cells, was comparable to that found in control tumors. At the end of the experiment, when the MTV reached 1.366 ± 0.098 cm^3^, all the mice-bearing IL30-overexpressing tumors had developed lung metastasis, versus just half of the control mice (Fisher’s exact test: *p* = 0.03; Fig. 4M), which also survived longer (64 vs 57 days. Log-rank test: *p* = 0.0012; Fig. 4N).

Since tumor overproduction of IL30 promoted a substantial intra-tumoral myeloid cell infiltrate**,** in both xenograft and syngeneic models, and in the latter also an influx of Tregs, whereas the genetic deletion of IL30 in human PC cells dampened inflammation in the tumor microenvironment, we wondered whether IL30 interfered with the cancer-immune cell crosstalk by regulating PC cell expression of immunity genes driving immune cell recruitment.

### Human membrane-bound and murine-secreted Interleukin-30 regulates the PC inflammation and immunity program

**Fig. 5:**
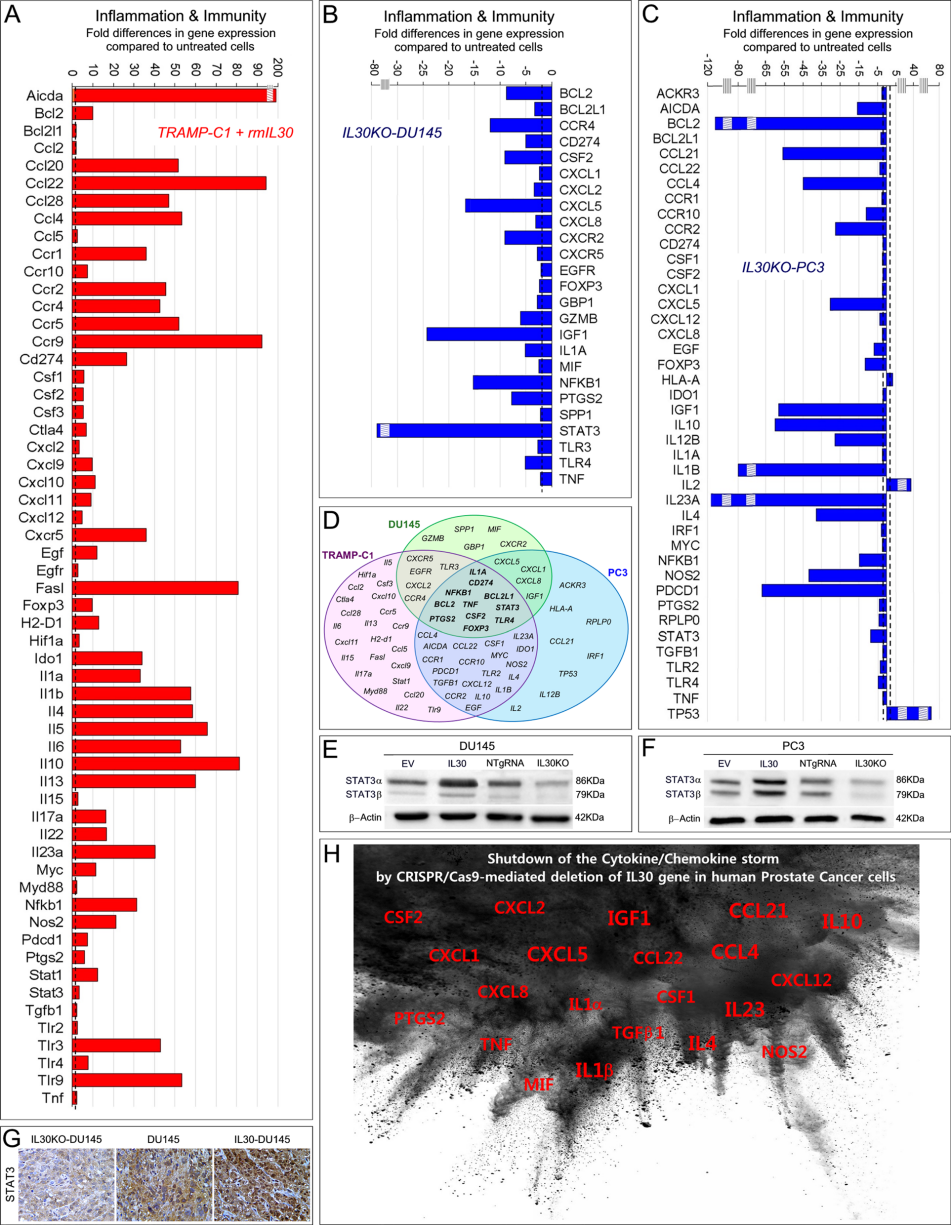
IL30-dependent regulation of inflammation and immunity gene expression in murine and human PC cells. **A** Mouse Cancer Inflammation & Immunity Crosstalk PCR Array. Fold differences of mRNAs of inflammation and immunity-related genes between rmIL30-treated (red bars) and untreated wild-type TRAMP-C1 cells. A significant threshold of twofold change in gene expression corresponded to *p* < 0.001. Only genes with a fold change > 2 are shown. Experiments were performed in duplicate. The stripped box represents a scale break. The dashed line represents the twofold change cutoff. **B** Human Cancer Inflammation & Immunity Crosstalk PCR Array. Fold differences of mRNAs of inflammation and immunity-related genes between IL30KO-DU145 cells (blue bars) and wild-type cells. Results obtained from control NTgRNA-treated DU145 cells were comparable to those from wild type cells. A significant threshold of a twofold change in gene expression corresponded to *p* < 0.001. Only genes with a fold change > 2 are shown. Experiments were performed in duplicate. The stripped box represents a scale break. The dashed line represents the twofold change cutoff. **C** Human Cancer Inflammation & Immunity Crosstalk PCR Array. Fold differences of mRNAs of inflammation and immunity-related genes between IL30KO-PC3 cells (blue bars) and wild-type cells. Results obtained from control NTgRNA-treated PC3 cells were comparable to those from wild-type cells. A significant threshold of a twofold change in gene expression corresponded to *p* < 0.001. Only genes with a fold change > 2 are shown. Experiments were performed in duplicate. Stripped boxes represent scale breaks. The dashed lines represent the twofold change cutoff. **D** Venn diagram representing the genes that govern “Cancer Inflammation & Immunity Crosstalk” which are up- and/or downregulated by IL30 (treatment with rmIL30 or human IL30 gene knockout) in TRAMP-C1 (purple circle), DU145 (green circle) and PC3 cells (blue circle). Overlapping circles illustrate the sharing of IL30-regulated genes between different cell lines. **E**, **F** Western blot analysis of the expression of STAT3 α and β isoforms in EV-, IL30 gene-, NTgRNA-treated and IL30KO-DU145 (**E**) and PC3 (**F**) cells**.** Results obtained from control NTgRNA-treated and EV-transfected cells were comparable to those from wild-type cells. **G** Immunostaining of STAT3 in tumors developed in NSG mice, after s.c. implantation of IL30 gene-transfected, or deleted, DU145 cells, revealed that the cytoplasmic and nuclear expression of STAT3 was strong in IL30-overexpressing tumors, scanty in IL30-deficient tumors and moderate in wild-type tumors. Immunostaining of control tumors, developed after implantation of EV-transfected or NTgRNA-treated cells, was comparable to that of wild-type tumors. Magnification: × 400. **H** Graphic representation of the suppression of cytokine and chemokine expression in DU145 and PC3 cells following IL30 gene deletion by CRISPR/Cas9 technology. The most downregulated molecular mediators are represented with a larger font size. Both IL23A and IL12B, which form the heterodimeric cytokine IL23, were suppressed

Inflammation and immunity gene expression profiles were investigated, by using PCR arrays, in TRAMP-C1 cells engineered to overexpress IL30 (Fig. 5A), and in DU145 and PC3 cells knocked out for the IL30 gene (Fig. 5B, C).

Activation-induced cytidine deaminase, also known as *AICDA*, which encodes a DNA-editing deaminase that mediates genotoxic effects [24] by enhancing the susceptibility to mutagenesis, was upregulated (193.72 times) in IL30-TRAMP-C1 versus control cells, along with expression of chemokines, such as *Ccl2* (2.01 times) [25], *Ccl4* (53.36 times) [26], *Ccl5* (2.56 times) [26] *and Cxcl2* (3.43 times) [27], and growth factors, such as *Csf1* (5.77 times) [27], *Csf2* (5.46 times) [28] and *Csf3* (5.46 times) [29], which promote MDC recruitment. IL30 overexpression in TRAMP-C1 cells, also led to a substantial upregulation (over 40 times increase) of C–C chemokines which drive Treg recruitment, such as *Ccl20* (51.55 times) [30], *Ccl22* (94.21 times) [31] and *Ccl28* (47.10 times) [32], and a storm of cytokines (more than 30 times increase) such as, *IL1a, IL1b, IL4, IL5, IL6, IL10, IL13, IL23a* and, to a lesser extent, *IL17a* and *IL22*. A significant upregulation (ranging from 9 to 11 times) of chemokines which may favor Treg, but also T and NK cell infiltrate, namely *Cxcl9, Cxcl10, Cxcl11* [33, 34] and *Cxcl12* [34] was also detected in IL30-TRAMP-C1 cells (Fig. 5A).

Intriguingly, in TRAMP-C1 cells, IL30 overproduction strongly upregulated the expression of chemokine receptors, namely *Ccr1* (35.95 times), *Ccr2* (45.50 times), *Ccr4* (42.75 times), *Ccr5* (51.90 times), *Ccr9* (92.27 times) [35] and *Cxcr5* (35.95 times) [36], which can promote cancer cell migration and metastatic spread.

In TRAMP-C1 cells, IL30 also boosted an immunosuppressive and tumor immune escape program (Fig. 5A), which includes the upregulation of *Ido1* (34.01 times), *Nos2* (21.37 times), *Ptgs2* (5.93 times) [37], *Cd274*, which encodes for *Pd-l1* (26.50 times), *Pdcd1*, which encodes for PD-1 (7.56 times), *Ctla4* (6.86 times) [38], *Tlr2-3-4-9* (2.51, 43.05, 7.61 and 53.36 times, respectively) [39] and, particularly, *FasL* (80.88 times) [40].

Assessment of the immunity gene expression profile in human PC cells, DU145, after IL30 gene deletion (Fig. 5B), revealed a substantial downregulation of oncogenes, such as *BCL2*, already included among the PC drivers, *BCL2L1, EGFR* [41] *and GBP1* [42], along with *SPP1, TLR3-4, TNF*, *IL1A* and particularly, *CSF2* (− 9.12 times) and *PTGS2* (− 7.78 times). Expression of *CD274/PD-L1*, along with a wide range of chemokines, mostly endowed with chemotactic activity toward macrophages and granulocytes, such as*, CXCL2/MIP2a* [43], *CXCL1* [44], *CXCL8* [45] *and MIF* [46], was also suppressed by IL30 gene deletion in DU145 cells.

In human PC cells, PC3, IL30 gene deletion led to a substantial suppression of a further range of proinflammatory mediators, primarily *CCL21* (− 56.00 times), *CCL4* (− 45.00 times), *IL1b* (− 80.00 times), *IL12B* (− 27.86 times), *IL23A* (− 115.00 times), *IL4* (− 38 times), *IL10* (− 60.00 times), whereas the expression of *IL2,* a pleiotropic cytokine with antimetastatic effects [47], was substantially upregulated (39.12 times). PC3 cells lacking constitutive IL30 expression also showed a considerable downregulation of *AICDA* (− 15.78), *FOXP3* (− 11.55) *NOS2* (− 42.00 times) and *PDCD1/PD-*1 (− 67.00 times), whereas the tumor suppressor, *TP53,* was remarkably upregulated (76.11 times) (Fig. 5C).

In both human PC cell lines, DU145 and PC3, IL30 gene deletion led to a significant downregulation of chemokine receptors, which promote cancer cell migration and metastatic dissemination [48] namely *CXCR5* (− 2.81 times), *CCR4* (− 11.96 times) and *CXCR2* (− 9.07 times) in DU145 cells, and *CCR1* (− 2.71 times), *CCR10* (− 11.00 times) and *CCR2* (− 27.67 times) in PC3 cells (Fig. 5B, C, D).

In addition to the suppression of *IGF1*, *BCL2* and *NFKB1*, disclosed by the analysis of PC driver genes, abrogation of endogenous IL30, consistently inhibited, in both human PC cell lines, the expression of STAT3, as confirmed at the protein level by WB analysis (Fig. 5E, F) and by immunohistochemistry in IL30KO tumors (Fig. 5G), and the expression of mediators of monocyte/macrophages and granulocyte recruitment, such as *CSF2* (28), *CXCL1* [44], *CXCL8* [45], *IL1A* [49], *TNF* [50] and, primarily *CXCL5* (− 16.96 times in IL30KO-DU145, and − 29.50 times in IL30KO-PC3) (Fig. 5H). Higher serum levels of CXCL5 were detected in metastatic PC patients, compared to patients with primary tumor or healthy subjects [51, 52]. The prominent regulation of CXCL5 expression by IL30, led us to speculate on its involvement in IL30 shaped PC progression program.

### CXCL5 autocrine loop contributes to IL30 driven proliferation of human PC cells

**Fig. 6:**
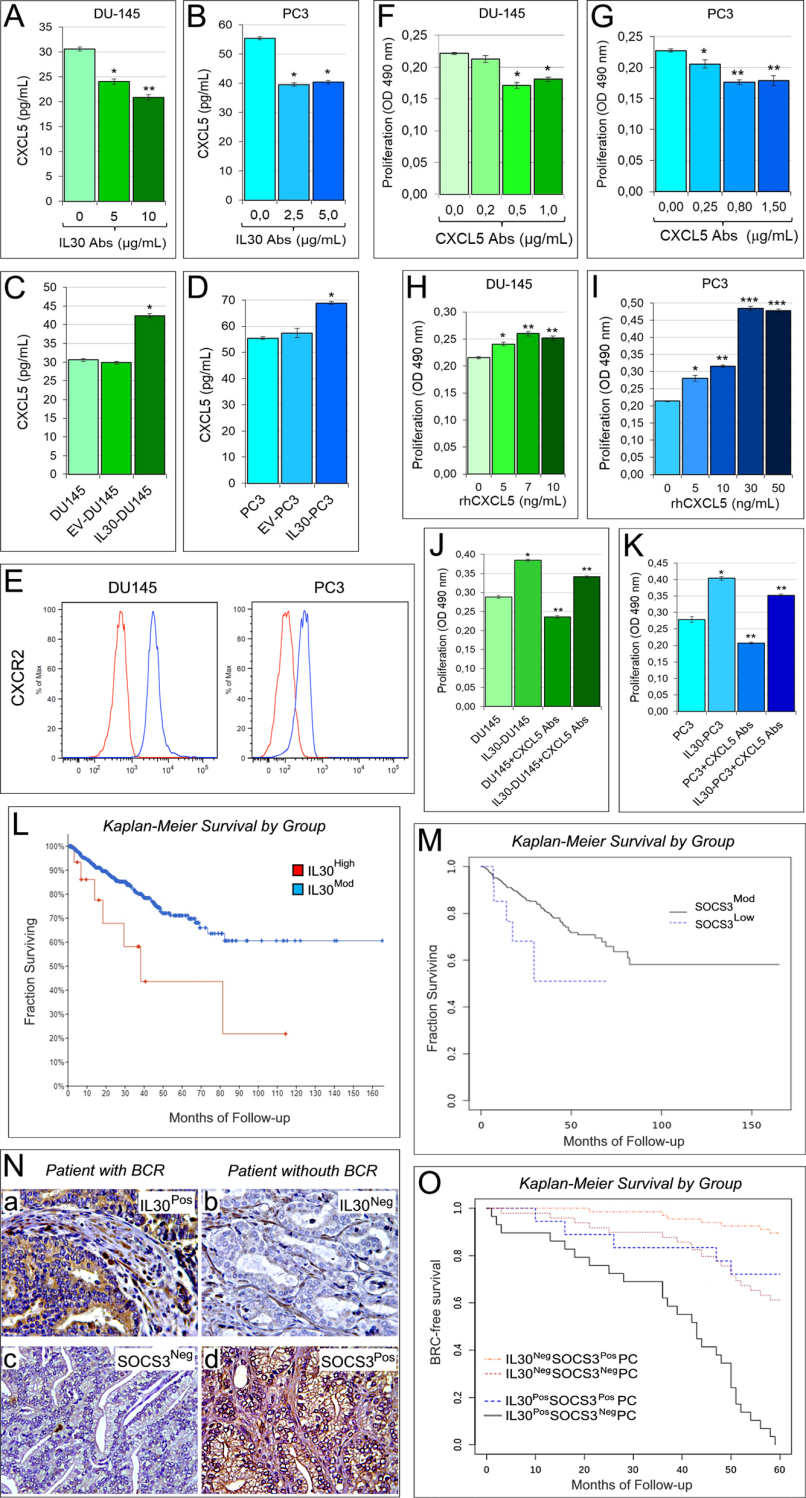
IL30-dependent regulation of CXCL5 production in human PC cells and survival curves of PC patients based on the expression of IL30 and SOCS3 in their clinical samples. **A**, **B** Elisa assay of CXCL5 release by wild type DU145 (**A**) and PC3 (**B**) cells, after the treatment with anti-IL30 Abs. **A** ANOVA: *p* < 0.0001. *p < 0.01, Tukey HSD test compared with untreated DU145 cells. **p < 0.01, Tukey HSD test compared with DU145 cells untreated or treated with 5 μg/mL. **B** ANOVA: *p* < 0.001. *p < 0.01, Tukey HSD test compared with untreated PC3 cells. Results are expressed as mean ± SD. **C**, **D** Elisa assay of CXCL5 release by wild type, EV- and IL30 gene-transfected DU145 (**C**) and PC3 (**D**) cells**.** ANOVA: *p* < 0.0001. *p < 0.01, Tukey HSD test compared with WT and EV-transfected DU145 cells. Results are expressed as mean ± SD. **E** Cytofluorimetric analyses of CXCR2 expression in PC3 and DU145 cells. Red lines: isotype control. Experiments were performed in triplicate. **F**, **G** MTT assay of DU145 (**F**) and PC3 (**G**) cells, untreated (0.0 μg/mL) or treated with anti-CXCL5 Abs (0.2, 0.5, 1.0 μg/mL in DU145; 0.25, 0.80, 1.50 μg/mL in PC3). (**F**) ANOVA: *p* < 0.0001. *p < 0.01, Tukey HSD test compared with 0.0 and 0.2 μg/mL. (G) ANOVA: *p* < 0.0001. *p < 0.01, Tukey HSD test compared with 0.0 µg/mL. **p < 0.01, Tukey HSD test compared with 0.0 and 0.25 μg/mL. Results are expressed as mean ± SD. **H**, **I** MTT assay of DU145 (**H**) and PC3 (**I**) cells, untreated (0.0 ng/mL) or treated with rhCXCL5 (5, 7, 10, ng/mL in DU145; 5, 10, 30, 50 ng/mL in PC3). **H** ANOVA: *p* < 0.0001. *p < 0.01, Tukey HSD test compared with 0 ng/mL. **p < 0.05, Tukey HSD test compared with 0 and 5 ng/mL. **I** ANOVA: *p* < 0.0001. *p < 0.01, Tukey HSD test compared with 0 ng/mL. **p < 0.01, Tukey HSD test compared with 0 and 5 ng/mL. ***p < 0.01, Tukey HSD test compared with 0, 5 and 10 ng/mL. Results are expressed as mean ± SD. **J**, **K** MTT assay of wild type and IL30 gene-transfected DU145 (**J**) and PC3 (**K**) cells, untreated (0.0 μg/mL), or treated with anti-CXCL5 Abs (0.5 μg/mL and 0.80 μg/mL, respectively). ANOVA: *p* < 0.0001. *p < 0.01, Tukey HSD test compared with wild type cells. **p < 0.01, Tukey HSD test compared with wild type and IL30 gene-transfected cells. Results are expressed as mean ± SD. **L** Kaplan–Meier curves representing, for each time point, the fraction of surviving PC patients, from the PanCancer collection, classified, based on mRNA expression levels in tumor samples, as IL30 mRNA^High^ (n.16/494) and IL30 mRNA^Mod^ (n.478/494). Log-rank test, *p* = 0.01. **M** Kaplan–Meier curves representing, for each time point, the fraction of surviving PC patients, from the PanCancer collection, classified, based on mRNA expression levels in tumor samples, as SOCS3 mRNA^Mod^ (n.476/493) and SOCS3 mRNA^Low^ (n.17/493). Log-rank test, *p* = 0.04. **N** Expression of IL30 (a, b) and SOCS3 (c, d) in PC tissues obtained from patients with (IL30^Pos^SOCS3^Neg^) or without (IL30^Neg^SOCS3^Pos^) biochemical recurrence (BCR). Magnification: × 630. **O** Kaplan–Meier curves representing, for each time point, the fraction of surviving PC patients classified, based on IL30 and SOCS3 expression in both tumor cells and infiltrating leukocytes, as IL30^Pos^SOCS3^Neg^PC (n.29), IL30^Pos^SOCS3^Pos^PC (n.18), IL30^Neg^SOCS3^Neg^PC (n.49) and IL30^Neg^SOCS3^Pos^PC (n. 67). Log-rank test, *p* < 0.001

Through its interaction with C-X-C chemokine receptor type 2 (CXCR2), CXCL5, also known as epithelial cell-derived neutrophil-activating peptide-78 (ENA-78), regulates neutrophil influx into the inflamed tissues, and stimulates tumor growth and progression directly or via infiltration and activation of granulocytes and MDCs [51, 53, 54]. Here, we asked whether CXCL5 was involved in IL30-dependent PC cell proliferation. By ELISA assay, we found that both DU145 and PC3 cells constitutively expressed and released CXCL5, 30.62 ± 0.38 and 55.37 ± 0.54 pg/mL, respectively. A reduced secretion of CXCL5 was demonstrated, by ELISA (ANOVA: *p* < 0.0001; Tukey HSD test: *p* < 0.01), after the blockade of endogenous IL30 with anti-IL30 Abs, 20.90 ± 0.54 pg/mL in DU145, and 39.53 ± 0.54 pg/mL in PC3 cells (Fig. 6A, B), whereas IL30 gene-transfected IL30-DU145 and IL30-PC3 cells produced and released higher levels of CXCL5 (42.40 ± 0.59 and 68.87 ± 0.54 pg/mL, respectively) than control cells (EV-DU145: 29.84 ± 0.33 pg/mL, EV-PC3: 57.37 ± 1.78 pg/mL; ANOVA: *p* < 0.0001; Tukey HSD test: *p* < 0.01) (Fig. 6C, D).

Both human PC cell types expressed CXCR2 (Fig. 6E), and the treatment with neutralizing anti-CXCL5 Abs resulted in a substantial (ANOVA: *p* < 0.0001; Tukey HSD test: *p* < 0.01) suppression of their proliferation (Fig. 6F, G) that was significantly improved (ANOVA: *p* < 0.0001; Tukey HSD test: *p* < 0.01) by the treatment with rhCXCL5 (Fig. 6H, I). Notably, the increased proliferation of IL30-overexpressing human PC cells versus controls, was inhibited, at least in part, by the treatment with anti-CXCL5 Abs (Fig. 6J, K). Therefore, CXCL5 exhibits autocrine activity and constitutes a novel pathway whereby IL30 may regulate human PC cell viability.

### The combination of low IL30 expression and high SOCS3 expression in clinical PC samples predicts a longer progression-free survival in PC patients

Here, we demonstrate that IL30 inhibits the expression of tumor suppressor SOCS3 in both murine and human PC cells, whereas SOCS3 was upregulated following genomic deletion of IL30 in human PC cells (Fig. 2F, G, H, I, L)**.** Epigenetic silencing of SOCS3 has been associated with high Gleason grade [55], and locally advanced PC [56], which more frequently express elevated levels of IL30 [3].

To assess the translational impact of SOCS3 regulation by IL30 expression in human PC cells, we analyzed RNA-Seq data of tumor samples from 494 PC patients, included the “*Prostate Adenocarcinoma TCGA PanCancer”* collection, along with their clinicopathological profiles (Table1).

Since the expression of IL30 was never below a *Z*-score = − 2, it was defined as ***high*** when the *Z* score was ≥ 2, and ***moderate*** when the *Z*-score was < 2. Since in the same tumor samples, the expression of SOCS3 was never higher than a *Z*-score = 2, it was defined as ***low*** when the *Z*-score was ≤ − 2, *and ****moderate*** when the *Z*-score was > − 2. Bioinformatics revealed that, irrespective of the grade and stage of the disease, patients bearing tumors with a ***high*** expression of ***IL30*** (IL30^High^; 16/494) had a median progression-free survival (PFS) of 38.20 months, whereas more than 50% of patients bearing tumors with a **moderate *IL30*** expression (IL30^Mod^; 478/494) were still progression free at the time of the last observation (166 months) (log-rank test, *p* = 0.01. Fig. 6L).

Moreover, patients bearing tumors with a ***low*** expression of ***SOCS3*** (SOCS3^Low^; 17/493) had a median PFS of 29.39 months, whereas more than 50% of patients bearing tumors with a ***moderate SOCS3*** expression (SOCS3^Mod^; 476/493) were still progression free at the time of the last observation (166 months) (log-rank test, *p* = 0.04. Fig. 6M).

To assess at the protein expression level, whether there was a correlation between IL30 and SOCS3 expression, we next selected from our institutional biobank a cohort of patients based on IL30 expression in their clinical samples. Since we reported that IL30 expression is tightly linked with advanced PC grade and stage (4), to obtain a cohort of patients in which the number of PC patients with a high level of expression of IL30 was suitable to provide an adequate confidence level (85% power at a 5% significance level), we selected 198 PC patients with Gleason score from 8 to 10 (high-grade tumors) and stage III disease (high-risk, clinically localized PC).

After staining for IL30, we applied the evaluation criteria previously described [7], and selected PC samples with or without IL30 expression, in both tumor cells and infiltrating leukocytes (IL30^Pos^PC, n. 52, and IL30^Neg^PC, n. 123, respectively).

Morphometric analysis revealed that 29 out of 52 patients (56%) with IL30^Pos^PC were also SOCS3^Neg^, whereas 18 patients (35%) were SOCS3^Pos^ (5 out of 52, 10%, were neither SOCS3^Pos^ nor SOCS3^Neg^).

Among the 123 patients diagnosed with IL30^Neg^PC, 49 (40%), were also SOCS3^Neg^, whereas 67 (54%) were SOCS3^Pos^ (7 out of 123, 6%, were neither SOCS3^Pos^ nor SOCS3^Neg^). These data demonstrated a significant (Chi-squared test, *p* = 0.0242) inverse association between IL30 and SOCS3 expression in PC samples (Fig. 6N).

Kaplan–Meier survival curves showed that all patients with IL30^Pos^SOCS3^Neg^PC (100%) underwent BCR, with a median BCR-free survival of 43 months, whereas only 5 out of 18 of patients (28%) bearing IL30^Pos^SOCS3^Pos^PC experienced BCR (Log-rank test, *p* < 0.0001) (Fig. 6O).

Kaplan-Meier analysis also showed that 19 out of 49 patients (39%) with IL30 ^Neg^SOCS3^Neg^PC underwent BCR, whereas only 7 out of 67 patients (10%) diagnosed with IL30^Neg^SOCS3^Pos^PC experienced BCR (Log-rank test, *p* = 0.0002) (Fig. 6O). Furthermore, the BCR-free survival of patients with IL30^Pos^SOCS3^Neg^PC was significantly lower than that of patients bearing IL30^Neg^SOCS3^Pos^PC (Log-rank test, *p* < 0.0001) (Fig. 6O).

Overall, the immunopathological studies related to the clinical outcome, substantiated the RNA-Seq data provided by the PanCancer dataset, and strengthened the clinical value of IL30’s regulation of SOCS3 expression in PC.

## Discussion

PC onset and progression stem from a complex interplay between genetic and epigenetic aberrations and microenvironmental factors, which dictate tumor behavior and clinical outcome [57]. Here, we demonstrate that in addition to shaping the immune gene expression profile, as we recently demonstrated in human breast cancer cells [14], membrane-bound IL30, which is constitutively expressed by human PC cells, regulates proliferation, invasion, migration and a wide range of oncogenic drivers of PC [57]. Parallel investigation of the regulatory effects of IL30 released by murine PC cells on their own transcriptional profile highlights that the human membrane-bound cytokine and its murine, albeit secreted, counterpart share essential biological functions. Via juxtacrine signaling, which clearly involves the phosphorylation and activation of the STAT1/STAT3 pathway [6, 19], as we previously described in murine PC cells [4], membrane-bound expression of IL30 on human PC cells sustains their proliferation, migration and invasion abilities. Furthermore, plasma membrane-anchored IL30 may orchestrate the genetic and immunological reprogramming of neighboring cancer cells by boosting the expression of oncogenes, growth factors, chemokine receptors, inflammatory mediators and checkpoint regulators, while turning off tumor suppressor genes.

Murine PC cells share a significant IL30-dependent upregulation of oncogenes, such as *Abcb1a*, that confers resistance to chemotherapy [58], *Bcl2*, that rescues cancer cells from apoptosis and drives androgen-independent growth [59], *Cav2*, which can activate cellular mitogenesis [60], and *Creb1*, which regulates a network of genes required for cell survival, proliferation and migration [61]. In murine PC cells, IL30 also promotes the expression of *Nfkb1*, the master regulator of immunity genes, cell-cycle modulators, survival signals, and of growth and angiogenic factors [62]; *Rbp1*, which regulates retinoic acid (RA) homeostasis, and that is silenced in more than 40% of PCs [63]; *Shbg*, which regulates testosterone metabolism and supports cell growth [64]; and *Tmprss2*, a membrane-anchored serine protease involved in PC progression, which can be found as *TMPRSS2-ERG* fusion gene in about 50% of PCs [65], and *Vegfa*, which promotes angiogenesis and metastasis [66]. Concurrently, IL30 overproduction led, in murine PC cell lines, to the downregulation of *Gstp1*, a member of the GST family of enzymes, that can protect cells from oxidative stress and has been found to be silenced in 90–95% of PCs [67].

Noteworthy, in both murine and human PC cells, IL30 stands out as an upstream regulator of key drivers of inflammation, immunity and cancer, such as *NFKB1* and *BCL2*, which in human PC cells can be efficiently suppressed by CRISPR/Cas9-mediated IL30 gene deletion. Targeted genome editing to abrogate constitutive production of IL30 also led, in both AR^+^ and AR^−^ human PC cell types, to the downregulation of a wide range of oncogenes, including AR, the main driver of castration-resistant PC development and of acquired resistance to androgen deprivation therapy, and *ERG*, which is overexpressed in a high proportion of PCs, due to a gene fusion with the androgen-driven promoter of the *TMPRSS2* gene [68]. In human PC cells, essential PC driver genes were suppressed following *IL30* gene knockout, such as *MKI67*, which is an established marker of tumor proliferation and an independent predictor of PC death [69], *FASN*, which modulates PC cell adhesion and migration and has been associates with BCR [70, 71], *HMGCR*, which is elevated in enzalutamide-resistant PC cells and has been associated with poor prognosis [72], and *PTGS2/Cox-2*, which catalyzes the rate-limiting steps in prostaglandin biosynthesis and could promote tumor growth and suppress tumor immunity [73]. Finally, the production of *IGF1*, which is heavily implicated in the pathogenesis of PC [20–23], can be massively inhibited in human PC cells by *IL30* gene deletion. IL30 regulates PC cell release of IGF1, which was increased by IL30 overexpression and suppressed by neutralizing anti-IL30 antibodies. IGF1 exerts autocrine growth function and mediates IL30-dependent proliferation of human PC cells, which can be largely suppressed by the treatment with anti-IGF1 neutralizing antibodies.

Besides downregulating relevant PC drivers, IL30 gene deletion rehabilitates in both human PC cell types, the expression of critical tumor suppressor genes, which are usually silenced in PC, such as *DKK3*, a secreted glycoprotein shown to inhibit TGFβ signaling, and primarily *SOCS3*, which negatively regulates JAK/STAT signaling and prevents cancer cell proliferation, invasion and metastasis [55].

The clinical relevance of our experimental findings is substantiated by the Kaplan–Meier curves of PC patients, from the “*Prostate Adenocarcinoma TCGA PanCancer”* collection, which show a shorter PFS for patients diagnosed with *IL30 mRNA*^*High*^ tumor and patients with *SOCS3 mRNA*^*Low*^ tumor. The immunopathological and morphometric study of PC samples from patients with high-grade and locally advanced disease, which more frequently express IL30 in their clinical samples, determined a significant association between the expression of IL30 and the lack of expression of SOCS3 in the tumor tissues and revealed a higher percentage of patients undergoing progression among those diagnosed with IL30^Pos^SOCS3^Neg^PC, when compared with patients diagnosed with IL30^Neg^ SOCS3^Pos^PC.

Monitoring of tumor xenografts in mice implanted with human IL30 knockout PC cells, demonstrates that suppression of the constitutive IL30 production slows tumor progression, reduces lung metastases and prolongs survival, whereas concomitant immunopathological analyses confirmed in vivo the downregulation of oncogenes, such as PTGS2 [73] and the upregulation of tumor suppressors, such as CDH1/E-Cadherin [74], PTEN [75], RARB [76], DKK3 [77] and SOCS3 [55, 56]. Intriguingly, in IL30 knockout tumor xenografts, overexpression of SOCS3 also involves infiltrating macrophage-like cells, suggesting that the effects of IL30 gene deletion in PC cells span to the surrounding immune cells. An overall quenching of microenvironmental inflammation in IL30 gene knockout tumors was demonstrated by the lack of myeloid cell infiltrate, which increased in IL30-overexpressing tumors. This immune cell context was confirmed in a fully immunocompetent murine PC model, in which, in addition to the recruitment of macrophages, MDCs and granulocytes, IL30 overproduction led to the intra-tumoral influx of Tregs, as also found in both experimental and clinical tissue samples of IL30-overexpressing PC [7], and likely promoted by the upregulation of PC cell expression of *Ccl4* [78], *Ccl20* [30], *Ccl22* [31] and *Ccl28* [32], which attract Tregs. Accordingly, in IL30 knockout human PC cells, the expression of CCL4 and CCL22 was substantially downregulated, along with the expression of IL12B and IL23A, coding for IL23 that is regulated by IL30 in murine and human PC cells, but also in murine and human breast cancer stem cells resulting in autocrine and paracrine effects [17].

The inflammatory landscape orchestrated by IL30 includes a wide range of immunomodulatory molecules, primarily cytokines, such as IL1, IL5, IL6, IL13 and IL17, which can shape the immune cell context and exert a variety of tumor-promoting functions [62], but also IL10 and IL4 both paradoxically endowed with pro- or anti-inflammatory effects depending on their sources, doses and timing of release, as well as the molecular and cellular environments [79, 80].

By contrast, the paucity of the lymphocyte infiltrate, in the immunocompetent PC model, despite the IL30 induced upregulation of T and NK chemoattractants, such as *Cxcl9*, *Cxcl10* and *Cxcl11*, could be attributed to immune evasion mechanisms, such as the upregulation of cancer cell expression of *FasL*, which induces lymphocyte apoptosis [81], and the upregulation of immune checkpoints, such as *Cd274/Pd-l1* [82] and *Cd152/Ctla4* [38], which by binding to their co-receptors on T cells, promote immune evasion and tumor progression, primarily through inhibition of cytotoxic T lymphocyte effector function.

Tumor progression and metastasis, and consequent reduced survival of mice-bearing IL30-overexpressing tumors, could be favored by the prominent upregulation of chemokine receptor expression [35, 48] in murine PC cells, such as *Ccr2, Ccr4, Ccr5, Ccr9* and *Cxcr5*. Consistently, the dramatic downregulation of *CCR2, CCR4, CXCR5, CCR1 and CCR10,* obtained by CRISPR/Cas9-mediated deletion of IL30, can cooperate in hindering disease progression in IL30 knockout tumor-bearing xenograft.

In addition to the suppression of IGF1, BCL2 and NFKB1, abrogation of the expression of membrane-bound IL30, by CRISPR/Cas9 genome editing, consistently inhibited, in both human PC cell lines, the expression of STAT3, confirmed at the protein level, by WB and immunohistochemical analysis, and downregulated macrophage and granulocyte chemoattractants, such as *CSF2* [28], *CXCL1* [44], *CXCL8* [45], *IL1A* [49], *TNF* [50] and, primarily *CXCL5*. Known as epithelial-derived neutrophil-activating peptide 78, ENA-78, CXCL5 stimulates chemotaxis and activation of neutrophils and MDSCs, through interaction with CXCR2 [51], but it can also regulate the expression of tumor-associated genes, like *BAX*, *NDRG3*, *IL18*, *BCL2* and *CASP3*, and stimulate PC cell proliferation [83]. Here we demonstrate that, as well as IGF1, CXCL5 feeds an autocrine growth loop that contributes to IL30-dependent PC cell proliferation, which can be hindered by the treatment with neutralizing anti-CXCL5 antibodies.

While confirming the tumor-promoting function of IL30, in both murine syngeneic and human xenograft models of PC, our findings demonstrate the membrane-bound expression of IL30 in human PC cells and unveil the novel mechanisms underlying its ability to boost, via juxtacrine signaling, a complex tumor progression program, which includes downregulation of tumor suppressor genes, such as SOCS3, and upregulation of oncogenes and growth factors, primarily IGF1 and CXCL5. The successful application of genome editing in targeting IL30 gene, in both AR+ and AR− human PC cells, and the improved survival of tumor-bearing hosts, provides the proof of concept for the use of CRISPR/Cas9 genome editing of IL30 to counteract prostatic oncogenesis in both the androgen-responsive and unresponsive phases of disease progression.

## Supplementary Information

**Additional file 1.** Supplemental Methods and Table S1 (list of antibodies used in immunostaining).

**Additional file 2.** Supplemental Figure 1 (Neutralization of IGF1 by Human IGF-1 antibody) and 2 (A.B. Cytofluorimetric analyses of androgen receptor expression in human PC cells DU145 and PC3. C.D. Western blot analyses of STAT1 and STAT3 protein expression in IL30-DU145 and IL30-PC3 cells).

## Data Availability

All data generated or analyzed during this study are available from the corresponding author on reasonable request.

## References

[1] GBD 2017 Disease and Injury Incidence and Prevalence Collaborators (2018). Global, regional, and national incidence, prevalence, and years lived with disability for 354 diseases and injuries for 195 countries and territories, 1990–2017: a systematic analysis for the Global Burden of Disease Study 2017. Lancet.

[2] Vitkin N, Nersesian S, Siemens DR, Koti M (2019). The tumor immune contexture of prostate cancer. Front Immunol.

[3] Di Meo S, Airoldi I, Sorrentino C, Zorzoli A, Esposito S, Di Carlo E (2014). Interleukin-30 expression in prostate cancer and its draining lymph nodes correlates with advanced grade and stage. Clin Cancer Res.

[4] Sorrentino C, Ciummo SL, Cipollone G, Caputo S, Bellone M, Di Carlo E (2018). Interleukin-30/IL27p28 shapes prostate cancer stem-like cell behavior and is critical for tumor onset and metastasization. Cancer Res.

[5] Pflanz S, Timans JC, Cheung J, Rosales R, Kanzler H, Gilbert J, Hibbert L, Churakova T, Travis M, Vaisberg E (2002). IL-27, a heterodimeric cytokine composed of EBI3 and p28 protein, induces proliferation of naive CD4+ T cells. Immunity.

[6] Garbers C, Spudy B, Aparicio-Siegmund S, Waetzig GH, Sommer J, Hölscher C, Rose-John S, Grötzinger J, Lorenzen I, Scheller J (2013). An interleukin-6 receptor-dependent molecular switch mediates signal transduction of the IL-27 cytokine subunit p28 (IL-30) via a gp130 protein receptor homodimer. J Biol Chem.

[7] Sorrentino C, Yin Z, Ciummo S, Lanuti P, Lu LF, Marchisio M, Bellone M, Di Carlo E (2019). Targeting Interleukin(IL)-30/IL-27p28 signaling in cancer stem-like cells and host environment synergistically inhibits prostate cancer growth and improves survival. J Immunother Cancer.

[8] Cancer Genome Atlas Research Network (2015). The molecular taxonomy of primary prostate cancer. Cell.

[9] Kaplan-Lefko PJ, Chen TM, Ittmann MM, Barrios RJ, Ayala GE, Huss WJ, Maddison LA, Foster BA, Greenberg NM (2003). Pathobiology of autochthonous prostate cancer in a pre-clinical transgenic mouse model. Prostate.

[10] Jachetti E, Mazzoleni S, Grioni M, Ricupito A, Brambillasca C, Generoso L, Calcinotto A, Freschi M, Mondino A, Galli R (2013). Prostate cancer stem cells are targets of both innate and adaptive immunity and elicit tumor-specific immune responses. Oncoimmunology.

[11] Mazzoleni S, Jachetti E, Morosini S, Grioni M, Piras IS, Pala M, Bulfone A, Freschi M, Bellone M, Galli R (2013). Gene signatures distinguish stage-specific prostate cancer stem cells isolated from transgenic adenocarcinoma of the mouse prostate lesions and predict the malignancy of human tumors. Stem Cells Transl Med.

[12] Foster BA, Gingrich JR, Kwon ED, Madias C, Greenberg NM (1997). Characterization of prostatic epithelial cell lines derived from transgenic adenocarcinoma of the mouse prostate (TRAMP) model. Cancer Res.

[13] Sorrentino C, Musiani P, Pompa P, Cipollone G, Di Carlo E (2011). Androgen deprivation boosts prostatic infiltration of cytotoxic and regulatory T lymphocytes and has no effect on disease-free survival in prostate cancer patients. Clin Cancer Res.

[14] Sorrentino C, Ciummo SL, D'Antonio L, Lanuti P, Abrams SI, Yin Z, Lu LF, Di Carlo E (2021). Hindering triple negative breast cancer progression by targeting endogenous interleukin-30 requires IFNγ signaling. Clin Transl Med.

[15] Stone KR, Mickey DD, Wunderli H, Mickey GH, Paulson DF (1978). Isolation of a human prostate carcinoma cell line (DU 145). Int J Cancer.

[16] Tai S, Sun Y, Squires JM, Zhang H, Oh WK, Liang CZ, Huang J (2011). PC3 is a cell line characteristic of prostatic small cell carcinoma. Prostate.

[17] Sorrentino C, Ciummo SL, D'Antonio L, Fieni C, Lanuti P, Turdo A, Todaro M, Di Carlo E (2021). Interleukin-30 feeds breast cancer stem cells via CXCL10 and IL23 autocrine loops and shapes immune contexture and host outcome. J Immunother Cancer.

[18] Müller SI, Friedl A, Aschenbrenner I, Esser-von Bieren J, Zacharias M, Devergne O, Feige MJ (2019). A folding switch regulates interleukin 27 biogenesis and secretion of its α-subunit as a cytokine. Proc Natl Acad Sci USA.

[19] Cheon H, Yang J, Stark GR (2011). The functions of signal transducers and activators of transcriptions 1 and 3 as cytokine-inducible proteins. J Interferon Cytokine Res.

[20] Koutsilieris M, Mitsiades C, Sourla A (2000). Insulin-like growth factor I and urokinase-type plasminogen activator bioregulation system as a survival mechanism of prostate cancer cells in osteoblastic metastases: development of anti-survival factor therapy for hormone-refractory prostate cancer. Mol Med.

[21] Heidegger I, Massoner P, Sampson N, Klocker H (2015). The insulin-like growth factor (IGF) axis as an anticancer target in prostate cancer. Cancer Lett.

[22] Chan JM, Stampfer MJ, Giovannucci E, Gann PH, Ma J, Wilkinson P, Hennekens CH, Pollak M (1998). Plasma insulin-like growth factor-I and prostate cancer risk: a prospective study. Science.

[23] Travis RC, Appleby PN, Martin RM, Holly JMP, Albanes D, Black A, Bueno-de-Mesquita HBA, Chan JM, Chen C, Chirlaque MD (2016). A Meta-analysis of individual participant data reveals an association between circulating levels of IGF-I and prostate cancer risk. Cancer Res.

[24] Stavnezer J (2011). Complex regulation and function of activation-induced cytidine deaminase. Trends Immunol.

[25] Chun E, Lavoie S, Michaud M, Gallini CA, Kim J, Soucy G, Odze R, Glickman JN, Garrett WS (2015). CCL2 Promotes colorectal carcinogenesis by enhancing polymorphonuclear myeloid-derived suppressor cell population and function. Cell Rep.

[26] Blattner C, Fleming V, Weber R, Himmelhan B, Altevogt P, Gebhardt C, Schulze TJ, Razon H, Hawila E, Wildbaum G (2018). CCR5+ myeloid-derived suppressor cells are enriched and activated in melanoma lesions. Cancer Res.

[27] Veglia F, Sanseviero E, Gabrilovich DI (2021). Myeloid-derived suppressor cells in the era of increasing myeloid cell diversity. Nat Rev Immunol.

[28] Bayne LJ, Beatty GL, Jhala N, Clark CE, Rhim AD, Stanger BZ, Vonderheide RH (2012). Tumor-derived granulocyte-macrophage colony-stimulating factor regulates myeloid inflammation and T cell immunity in pancreatic cancer. Cancer Cell.

[29] Bruno A, Mortara L, Baci D, Noonan DM, Albini A (2019). Myeloid derived suppressor cells interactions with natural killer cells and pro-angiogenic activities: roles in tumor progression. Front Immunol.

[30] Wang D, Yang L, Yu W, Wu Q, Lian J, Li F, Liu S, Li A, He Z, Liu J (2019). Colorectal cancer cell-derived CCL20 recruits regulatory T cells to promote chemoresistance via FOXO1/CEBPB/NF-κB signaling. J Immunother Cancer.

[31] Klarquist J, Tobin K, Farhangi Oskuei P, Henning SW, Fernandez MF, Dellacecca ER, Navarro FC, Eby JM, Chatterjee S, Mehrotra S (2016). Ccl22 diverts T regulatory cells and controls the growth of melanoma. Cancer Res.

[32] Ji L, Qian W, Gui L, Ji Z, Yin P, Lin GN, Wang Y, Ma B, Gao WQ (2020). Blockade of β-catenin-induced CCL28 suppresses gastric cancer progression via inhibition of Treg cell infiltration. Cancer Res.

[33] Kohli K, Pillarisetty VG, Kim TS (2022). Key chemokines direct migration of immune cells in solid tumors. Cancer Gene Ther.

[34] Susek KH, Karvouni M, Alici E, Lundqvist A (2018). The role of CXC chemokine receptors 1–4 on immune cells in the tumor microenvironment. Front Immunol.

[35] Jacquelot N, Duong CPM, Belz GT, Zitvogel L (2018). Targeting chemokines and chemokine receptors in melanoma and other cancers. Front Immunol.

[36] Kazanietz MG, Durando M, Cooke M (2019). CXCL13 and its receptor CXCR5 in cancer: inflammation, immune response, and beyond. Front Endocrinol (Lausanne).

[37] Davila-Gonzalez D, Chang JC, Billiar TR (2017). NO and COX2: dual targeting for aggressive cancers. Proc Natl Acad Sci U S A.

[38] Seidel JA, Otsuka A, Kabashima K (2018). Anti-PD-1 and anti-CTLA-4 therapies in cancer: mechanisms of action, efficacy, and limitations. Front Oncol.

[39] Javaid N, Choi S (2020). Toll-like receptors from the perspective of cancer treatment. Cancers (Basel).

[40] Kim R, Emi M, Tanabe K, Uchida Y, Toge T (2004). The role of Fas ligand and transforming growth factor beta in tumor progression: molecular mechanisms of immune privilege via Fas-mediated apoptosis and potential targets for cancer therapy. Cancer.

[41] Nastały P, Stoupiec S, Popęda M, Smentoch J, Schlomm T, Morrissey C, Żaczek AJ, Beyer B, Tennstedt P, Graefen M (2020). EGFR as a stable marker of prostate cancer dissemination to bones. Br J Cancer.

[42] Honkala AT, Tailor D, Malhotra SV (2020). Guanylate-binding protein 1: an emerging target in inflammation and cancer. Front Immunol.

[43] Capucetti A, Albano F, Bonecchi R (2020). Multiple roles for chemokines in neutrophil biology. Front Immunol.

[44] De Filippo K, Dudeck A, Hasenberg M, Nye E, van Rooijen N, Hartmann K, Gunzer M, Roers A, Hogg N (2013). Mast cell and macrophage chemokines CXCL1/CXCL2 control the early stage of neutrophil recruitment during tissue inflammation. Blood.

[45] Liu Q, Li A, Tian Y, Wu JD, Liu Y, Li T, Chen Y, Han X, Wu K (2016). The CXCL8-CXCR1/2 pathways in cancer. Cytokine Growth Factor Rev.

[46] Calandra T, Roger T (2003). Macrophage migration inhibitory factor: a regulator of innate immunity. Nat Rev Immunol.

[47] Jiang T, Zhou C, Ren S (2016). Role of IL-2 in cancer immunotherapy. Oncoimmunology.

[48] Mollica Poeta V, Massara M, Capucetti A, Bonecchi R (2019). Chemokines and chemokine receptors: new targets for cancer immunotherapy. Front Immunol.

[49] Di Paolo NC, Shayakhmetov DM (2016). Interleukin 1α and the inflammatory process. Nat Immunol.

[50] Hickey MJ, Reinhardt PH, Ostrovsky L, Jones WM, Jutila MA, Payne D, Elliott J, Kubes P (1997). Tumor necrosis factor-alpha induces leukocyte recruitment by different mechanisms in vivo and in vitro. J Immunol.

[51] Zhang W, Wang H, Sun M, Deng X, Wu X, Ma Y, Li M, Shuoa SM, You Q, Miao L (2020). CXCL5/CXCR2 axis in tumor microenvironment as potential diagnostic biomarker and therapeutic target. Cancer Commun (Lond).

[52] Roca H, Jones JD, Purica MC, Weidner S, Koh AJ, Kuo R, Wilkinson JE, Wang Y, Daignault-Newton S, Pienta KJ (2018). Apoptosis-induced CXCL5 accelerates inflammation and growth of prostate tumor metastases in bone. J Clin Investig.

[53] Begley LA, Kasina S, Mehra R, Adsule S, Admon AJ, Lonigro RJ, Chinnaiyan AM, Macoska JA (2008). CXCL5 promotes prostate cancer progression. Neoplasia.

[54] Wang G, Lu X, Dey P, Deng P, Wu CC, Jiang S, Fang Z, Zhao K, Konaparthi R, Hua S (2016). Targeting YAP-dependent MDSC infiltration impairs tumor progression. Cancer Discov.

[55] Pierconti F, Martini M, Pinto F, Cenci T, Capodimonti S, Calarco A, Bassi PF, Larocca LM (2011). Epigenetic silencing of SOCS3 identifies a subset of prostate cancer with an aggressive behavior. Prostate.

[56] Dai L, Li Z, Tao Y, Liang W, Hu W, Zhou S, Fu X, Wang X (2021). Emerging roles of suppressor of cytokine signaling 3 in human cancers. Biomed Pharmacother.

[57] Wang G, Zhao D, Spring DJ, DePinho RA (2018). Genetics and biology of prostate cancer. Genes Dev.

[58] Zhu Y, Liu C, Armstrong C, Lou W, Sandher A, Gao AC (2015). Antiandrogens inhibit ABCB1 efflux and ATPase activity and reverse docetaxel resistance in advanced prostate cancer. Clin Cancer Res.

[59] Lin Y, Fukuchi J, Hiipakka RA, Kokontis JM, Xiang J (2007). Up-regulation of Bcl-2 is required for the progression of prostate cancer cells from an androgen-dependent to an androgen-independent growth stage. Cell Res.

[60] Gould ML, Williams G, Nicholson HD (2010). Changes in caveolae, caveolin, and polymerase 1 and transcript release factor (PTRF) expression in prostate cancer progression. Prostate.

[61] Watson MJ, Berger PL, Banerjee K, Frank SB, Tang L, Ganguly SS, Hostetter G, Winn M, Miranti CK (2021). Aberrant CREB1 activation in prostate cancer disrupts normal prostate luminal cell differentiation. Oncogene.

[62] Staal J, Beyaert R (2018). Inflammation and NF-κB signaling in prostate cancer: mechanisms and clinical implications. Cells.

[63] Jerónimo C, Henrique R, Oliveira J, Lobo F, Pais I, Teixeira MR, Lopes C (2004). Aberrant cellular retinol binding protein 1 (CRBP1) gene expression and promoter methylation in prostate cancer. J Clin Pathol.

[64] Li H, Pham T, McWhinney BC, Ungerer JP, Pretorius CJ, Richard DJ, Mortimer RH, d'Emden MC, Richard K (2016). Sex hormone binding globulin modifies testosterone action and metabolism in prostate cancer cells. Int J Endocrinol.

[65] Yu J, Yu J, Mani RS, Cao Q, Brenner CJ, Cao X, Wang X, Wu L, Li J, Hu M (2010). An integrated network of androgen receptor, polycomb, and TMPRSS2-ERG gene fusions in prostate cancer progression. Cancer Cell.

[66] Botelho F, Pina F, Lunet N (2010). VEGF and prostatic cancer: a systematic review. Eur J Cancer Prev.

[67] Schnekenburger M, Karius T, Diederich M (2014). Regulation of epigenetic traits of the glutathione S-transferase P1 gene: from detoxification toward cancer prevention and diagnosis. Front Pharmacol.

[68] Adamo P, Ladomery MR (2016). The oncogene ERG: a key factor in prostate cancer. Oncogene.

[69] Kammerer-Jacquet SF, Ahmad A, Møller H, Sandu H, Scardino P, Soosay G, Beltran L, Cuzick J, Berney DM (2019). Ki-67 is an independent predictor of prostate cancer death in routine needle biopsy samples: proving utility for routine assessments. Mod Pathol.

[70] De Piano M, Manuelli V, Zadra G, Otte J, Edqvist PD, Pontén F, Nowinski S, Niaouris A, Grigoriadis A, Loda M (2020). Lipogenic signalling modulates prostate cancer cell adhesion and migration via modification of Rho GTPases. Oncogene.

[71] Cao Z, Xu Y, Guo F, Chen X, Ji J, Xu H, He J, Yu Y, Sun Y, Lu X (2020). FASN protein overexpression indicates poor biochemical recurrence-free survival in prostate cancer. Dis Mark.

[72] Kong Y, Cheng L, Mao F, Zhang Z, Zhang Y, Farah E, Bosler J, Bai Y, Ahmad N, Kuang S (2018). Inhibition of cholesterol biosynthesis overcomes enzalutamide resistance in castration-resistant prostate cancer (CRPC). J Biol Chem.

[73] Liu B, Qu L, Yan S (2015). Cyclooxygenase-2 promotes tumor growth and suppresses tumor immunity. Cancer Cell Int.

[74] Song MS, Carracedo A, Salmena L, Song SJ, Egia A, Malumbres M, Pandolfi PP (2011). Nuclear PTEN regulates the APC-CDH1 tumor-suppressive complex in a phosphatase-independent manner. J Cell.

[75] Jamaspishvili T, Berman DM, Ross AE, Scher HI, De Marzo AM, Squire JA, Lotan TL (2018). Clinical implications of PTEN loss in prostate cancer. Nat Rev Urol.

[76] Dou M, Zhou X, Fan Z, Ding X, Li L, Wang S, Xue W, Wang H, Suo Z, Deng X (2018). Clinical significance of retinoic acid receptor beta promoter methylation in prostate cancer: a meta-analysis. Cell Physiol Biochem.

[77] Kardooni H, Gonzalez-Gualda E, Stylianakis E, Saffaran S, Waxman J, Kypta RM (2018). CRISPR-mediated reactivation of DKK3 expression attenuates TGF-β Signaling in prostate cancer. Cancers (Basel).

[78] Schlecker E, Stojanovic A, Eisen C, Quack C, Falk CS, Umansky V, Cerwenka A (2012). Tumor-infiltrating monocytic myeloid-derived suppressor cells mediate CCR5-dependent recruitment of regulatory T cells favoring tumor growth. J Immunol.

[79] Skapenko A, Niedobitek GU, Kalden JR, Lipsky PE, Schulze-Koops H (2004). Generation and regulation of human Th1-biased immune responses in vivo: a critical role for IL-4 and IL-10. J Immunol.

[80] Ul-Haq Z, Naz S, Mesaik MA (2016). Interleukin-4 receptor signaling and its binding mechanism: a therapeutic insight from inhibitors tool box. Cytokine Growth Factor Rev.

[81] Anderson KG, Oda SK, Bates BM, Burnett MG, Rodgers Suarez M, Ruskin SL, Greenberg PD (2022). Engineering adoptive T cell therapy to co-opt Fas ligand-mediated death signaling in ovarian cancer enhances therapeutic efficacy. J Immunother Cancer.

[82] Hudson K, Cross N, Jordan-Mahy N, Leyland R (2020). The extrinsic and intrinsic roles of PD-L1 and its receptor PD-1: implications for immunotherapy treatment. Front Immunol.

[83] Qi Y, Zhao W, Li M, Shao M, Wang J, Sui H, Yu H, Shao W, Gui S, Li J (2018). High C-X-C motif chemokine 5 expression is associated with malignant phenotypes of prostate cancer cells via autocrine and paracrine pathways. Int J Oncol.

