# Supplementary material for: CRISPR/Cas9-mediated deletion of Interleukin-30 suppresses IGF1 and CXCL5 and boosts SOCS3 reducing prostate cancer growth and mortality
Source: J Hematol Oncol. 2022 Oct 13;15:145. doi: 10.1186/s13045-022-01357-6 (PMC9559017; doi:10.1186/s13045-022-01357-6)
Supplement: Supplementary file 1 — Additional file 1. Supplemental Methods and Table S1 (list of antibodies used in immunostaining). [file 13045_2022_1357_MOESM1_ESM.docx]

**Supplemental Methods**

**Flow cytometry**

For flow cytometric analysis, cells were incubated with the following antibodies (Abs): anti-human CD126 (BD Biosciences Cat# 551850, RRID:AB_394271); anti-mouse CD126 (Thermo Fisher Scientific Cat# 12-1261-80, RRID:AB_996658); anti-human CD130 (Abcam Cat# ab47218, RRID:AB_868803); anti-mouse CD130 (Thermo Fisher Scientific Cat# 17-1302-82, RRID:AB_10670874); anti-human IL30 (Thermo Fisher Scientific Cat# 50-8277-42, RRID:AB_11149127), anti-human CXCR2 (Miltenyi Biotec Cat# 130-126-197, RRID:AB_2889483) and anti-human IGF1R (Thermo Fisher Scientific Cat# 17-8849-41, RRID:AB_1907365).

**Transfection with *IL27p28* (*IL30*) expressing vector**

To generate the mouse *Il30* lentiviral expression vector, a 728 bp fragment, carrying the gene for *Il30*, was amplified from the cDNA of TRAMP-C1, using the Platinum Taq polymerase High Fidelity (#10966018; Thermo Fisher Scientific, Waltham, MA, USA) with the following primers: IL30 forward (5′-GCTCTAGAATGGGCCAGGTGACAG-3′) and IL30 reverse (5′-CCGCTCGAGTTAGGAATCCCAGGCT-3′).

The amplified fragment was cloned into XbaI-XhoI unique sites of a Tween lentiviral vector, under the control of a CMV promoter. This vector constitutively expresses GFP (Green Fluorescent Protein), under the control of a PGK promoter. Then, TRAMP-C1 cells were transfected with the p-TWEEN-mIL-30 or the empty vector p-TWEEN, using Calcium Phosphate Transfection Kit (#K278001; Thermo Fisher Scientific, Waltham, MA, USA).

The infected cells were sorted using a FACSAria II Cell Sorter (RRID:SCR_018934; BD Biosciences, Franklin Lakes, NJ, USA) using GFP as marker. Expression of IL30 was confirmed by real-time RT-PCR, Western Blotting (WB) and ELISA assay.

**CRISPR/Cas9-mediated IL30 gene** **knockout**

For CRISPR mediated *Il30* gene deletion in murine PC-SLCs, we used two guide (g)RNA sequences, designed and synthesized by GenScript (gRNA 1 and 2), which were cloned into the eSpCas9-2A-GFP (PX458) vector (GenScript, Piscataway, NJ, USA). Murine PC-SLCs were transfected with either gRNA 1 or 2, using Safectine RU50 (SydLabs, Hopkinton, MA, USA), to derive two independent clonal cell populations (clone 1 and 2). For CRISPR mediated IL30 gene deletion in human cell lines, we used two Trueguide Synthetic (s)gRNAs, designed and synthesized by Thermofisher (#CRISPR947384_SGM, IL27p28, Human and #CRISPR947272_SGM, IL27p28, Human). Cells were transfected with sgRNAs using CRISPRMAX™ Cas9 Transfection Reagent (#CMAX00001; Thermo Fisher Scientific, Waltham, MA, USA), to derive two independent clonal cell populations (clone A and B). Then, transfected cells were sorted with a BD FACSAria II Cell Sorter (RRID:SCR_018934), using the GFP as marker. Since WB and ELISA assay showed that the expression of IL30 protein was completely abolished both in murine cell clones 1 and 2 and in human cell clones A and B, we used only clone 1 and clone A in subsequent experiments. To exclude off-target effects, we performed genetic rescue experiments transfecting knock-out cell clones with IL30 expressing vector, which restored the proliferation rates prior to genome editing.

***STAT1* and *STAT3* knockdown experiments**

For the silencing of *STAT1* or *STAT3* genes in both DU145 and PC3 cell lines, we used the FlexiTube GeneSolution kit (#1027416, Qiagen, Hilden, Germany). Each kit is a gene-specific package of 4 preselected siRNAs designed to target human *STAT1* or *STAT3* gene. Each siRNA was transfected into PC3 and DU145 cells overexpressing IL30 (IL30-PC3 and IL30-DU145, respectively), using HiPerFect Transfection Reagent (Qiagen), according to the manufacturer’s instructions. To evaluate the gene-silencing efficiency, RNA was extracted 24 and 48 hours after cell transfection for real-time RT-PCR and the cell pellets were collected for WB. We selected the two siRNAs with the highest knockdown efficiency for each target and we used only these two siRNAs in subsequent experiments. The AllStars Negative Control siRNA (Qiagen) was used as a negative controls (scrambled siRNAs). To investigate the effect of *STAT1* or *STAT3* silencing on IL30-driven proliferation, migration and invasion, IL30-overexpressing PC3 (IL30-PC3) and DU145 (IL30-DU145) cells were seeded on 24-well plates and transfected with siRNAs which targeted *STAT1* or *STAT3*. Twenty-four hours after the transfection, cell proliferation, migration and invasion of *STAT1* or *STAT3* siRNAs transfected and untrasfected IL30-DU145 and IL30-PC3 cells were assessed using the CellTiter 96 AQueous One Solution Cell Proliferation Assay (#G3582; Promega, Madison, WI, USA) and the CytoSelect Cell Migration and Invasion Assay (#CBA-100-C; Cell Biolabs, San Diego, CA, USA), according to manufacturer’s instructions.

**PCR array and real-time RT-PCR**

RNA was extracted from human and murine PC cells by using the RNeasy Mini Kit (#74104, Qiagen, Hilden, Germany), and reverse-transcribed with the RT2 First Strand Kit (#330401, Qiagen). PCR array analyses were run on a Qiagen Rotor Gene Q (Qiagen Rotor-Gene Q, RRID:SCR_018976), using the RT2 SYBR Green ROX FAST Master mix (#330501) and the RT² Profiler Human Cancer Inflammation & Immunity Crosstalk PCR Array (#PAHS-181Z), the RT² Profiler™ Human Prostate Cancer PCR Array (#PAHS-135Z), the RT² Profiler Mouse Cancer Inflammation & Immunity Crosstalk PCR Array (#PAMM-181Z) and the RT² Profiler™ Mouse Prostate Cancer PCR Array (#PAMM-135Z) (all from Qiagen). The results from each plate were normalized to the median value of a set of housekeeping genes. Changes in the gene expression were calculated using the ΔΔCt method. Results from experiments performed in triplicate were pooled and analyzed with the manufacturer’s software. A significant threshold of a 2-fold change in gene expression corresponded to a *p*<0.001.

For analyses of IL30 mRNA levels in human and murine PC cells, real-time RT-PCR was performed with the Quantifast SYBR Green PCR Kit (#204054, Qiagen) and a MiniOpticon System (#CFB-3120, Bio-Rad, Hercules, CA, USA), using the following primers: Human_IL27p28_1_SG QuantiTect Primer Assay (#QT00236250) and Mouse_Il27p28_1_SG QuantiTect Primer Assay (QT00143017) (both from Qiagen).

**Western blotting**

For total protein extraction, cells were collected by centrifugation and lysed with ice cold RIPA Lysis buffer (Thermo Fisher Scientific, Waltham, MA, USA), supplemented with Protease and Phosphatase Inhibitors Cocktails (Thermo Fisher Scientific, Waltham, MA, USA). Total proteins were then quantified using the Bradford assay. For cytosolic and plasma membrane protein extraction, Minute Plasma Membrane Protein Isolation and Cell Fractionation Kit (#SM-005; Invent Biotechnologies, Plymouth, MN, USA) was used, according to manufacturer’s protocol. Cytosolic and plasma membrane fractions were then quantified using Pierce BCA Protein Assay Kit (#23225; Thermo Fisher Scientific, Waltham, MA, USA). Subsequently, whole cell lysates, cytosolic and plasma membrane fractions were loaded on Mini-PROTEAN TGX Gels 4-20% (#4561094; Bio-Rad, Hercules, CA, USA) and proteins were transferred from the gels on Immuno-Blot PVDF Membranes (#1620177; Bio-Rad, Hercules, CA, USA) in transfer buffer (glycine, tris [pH 8.4] and methanol) using Mini Trans-Blot Cell apparatus (Bio-Rad). Membranes containing the transferred proteins were then blocked with 5% milk (Sigma-Aldrich, St. Louis, MO, USA) in TBST and, subsequently, probed with primary and horseradish peroxidase conjugated secondary antibodies, following standard procedures. The following primary and secondary antibodies were used: rabbit anti-human IL-27-A (Abcam Cat# ab118910, RRID:AB_10898806); goat anti-mouse IL-27p28/IL30 (R&D Systems Cat# MAB7430, RRID:AB_11129241); rabbit anti-phospho-STAT1 (Cell Signaling Technology Cat# 9167, RRID:AB_561284); rabbit anti-phospho-STAT3 (Cell Signaling Technology Cat# 9145, RRID:AB_2491009); mouse anti-STAT3 (Cell Signaling Technology Cat# 9139, RRID:AB_331757); rabbit anti-BCL2 (BD Biosciences Cat# 554279, RRID:AB_395341); rabbit anti-human NFKB1 (Abcam Cat# ab32360, RRID:AB_776748); rabbit anti-human DKK3 (Thermo Fisher Scientific Cat# PA5-102626, RRID:AB_2852023); mouse anti-human SOCS3 (#ab236519; Abcam, Cambridge, UK); goat anti-rabbit IgG (H + L)-HRP Conjugate (Sigma-Aldrich Cat# A0545, RRID:AB_257896); rabbit anti-mouse IgG (whole molecule)-Peroxidase (Sigma-Aldrich Cat# A9044, RRID:AB_258431) and rabbit anti-goat IgG (whole molecule)-Peroxidase (Sigma-Aldrich Cat# A5420, RRID:AB_258242). β-Actin (Sigma-Aldrich Cat# A2228, RRID: AB_476697) and Sodium Potassium ATPase (Thermo Fisher Scientific Cat# MA5-32184, RRID:AB_2809472) were used as loading controls for total/cytosolic proteins and plasma membrane proteins, respectively. Membranes were then washed with TBST and developed with Pierce ECL Western Blotting Substrate (#32106; Thermo Fisher Scientific, Waltham, MA, USA).

**Histopathology, immunohistochemistry** **and morphometric analyses**

Immunostainings were performed using the antibodies listed in Table S1.

Proliferation index, microvessel and cell counts, on tumor samples derived from mouse models, were assessed by light microscopy, at ×400 in an 85,431.59 μm^2^ field, on single immunostained sections, using QWin image analysis software (Leica QWin, RRID:SCR_018940), which ensures the following highly reproducible steps: 1) image acquisition; 2) conversion of RGB image (true colors) to binary image (black and white); 3) filtering to remove noise; 4) counting of immunostained cells or measurement of positively stained area. Six to eight high-power fields were analyzed for each section and three sections per sample were evaluated. Results were expressed as mean ± SD of positive cells per field (F4/80, Ly-6G, CD11b/Gr-1, CD3, CD8, CD4, Foxp3), or mean percentage ± SD of positive cells/number of total cells per field (PCNA, Ki67). Microvessels were identified as small tubes or circles marked by CD31 Abs and results were expressed as mean ± SD of positive vessels/field.

The morphometric analysis on human PC samples was confined to the neoplastic areas of prostatic tissue sections, and was performed by light microscopy, at ×400, in an 85431.59 μm^2^ field, on single immunostained sections, with Qwin image analysis software (Qiagen) as described above. Results were expressed as mean percentage ± SD of (IL30 or SOCS3) positive cells/number of total cells per field.

**Immunoelectron microscopy**

For immunogold labeling of PC cells, ultrathin cryosections (55 nm) were cut at -110°C, using an Ultracut EM FC6 (Leica Microsystems, Wetzlar, Germany), collected with 1% methyl cellulose in 1.15 M sucrose, blocked in 1% bovine serum albumin in PBS and then immunolabeled with primary antibody, a rabbit polyclonal anti-IL27A (Abcam Cat# ab118910, RRID:AB_10898806). Bound antibodies were visualized using either goat anti-rabbit conjugated with 10 nm gold particles (#AC-10-01-05, Cytodiagnostics, Burlington, Ontario, Canada) or 10 nm PrA-CG (acquired from G. Posthuma, Utrecht, The Netherlands). All incubations were performed in presence of 1% BSA.

**Table S1.** Antibodies used in immunostaining

| **Antibody** | **Clone** | **Origin** | **Research Resource Identifiers (RRIDs)** | **Source** |
| --- | --- | --- | --- | --- |
|  |  |  |  |  |
| ***Anti-human*** |  |  |  |  |
| DKK3 |  | Rabbit | RRID:AB_2852023 | Thermo Fisher, Waltham, MA, USA |
| E-cadherin | NCH-38 | Mouse | RRID:AB_2076672 | Agilent, Santa Clara, CA, USA |
| IGF1 |  | Rabbit | RRID:AB_308724 | Abcam, Cambridge, UK |
| IL30 |  | Rabbit | RRID:AB_10898806 | “ |
| Ki67 | MIB1 | Mouse | RRID:AB_2142367 | Agilent, Santa Clara, CA, USA |
| NFKB1* | E381 | Rabbit | RRID:AB_776748 | Abcam, Cambridge, UK |
| PTEN | 28H6 | Mouse | RRID:AB_383709 | GeneTex, Hsinchu City, Taiwan |
| PTGS2 |  | Rabbit | RRID:AB_1951531 | “ |
| RARB |  | Rabbit | RRID:AB_11021140 | Novus Biologicals,  Centennial, CO, USA |
| SOCS3 | OTI3D3 | Mouse | Cat. n. ab236519 | Abcam, Cambridge, UK |
|  |  |  |  |  |
| ***Anti-mouse*** |  |  |  |  |
| CD3 |  | Rabbit | RRID:AB_2335677 | Agilent, Santa Clara, CA, USA |
| CD4 | YTS191.1 | Rat | RRID:AB_323559 | Bio-Rad, Hercules, CA, USA |
| CD8 | YTS169.4 | Rat | RRID:AB_322770 | “ |
| CD11b | EPR1344 | Rabbit | RRID:AB_2650514 | Abcam, Cambridge, UK |
| CD31 | SZ31 | Rat | RRID:AB_2631039 | Dianova, Hamburg, Germany |
| F4/80 | Cl:A3-1 | Rat | RRID:AB_323279 | Bio-Rad, Hercules, CA, USA |
| Foxp3 | FJK-16s | Rat | RRID:AB_467576 | Thermo Fisher, Waltham, MA, USA |
| Gr-1 | RB6-8C5 | Rat | RRID:AB_394638 | BD Biosciences,  Franklin Lakes, NJ, USA |
| IL30 |  | Goat | RRID: AB_355012 | R&D Systems,  Minneapolis, MN, USA |
| Ly-6G | 1A8 | Rat | RRID:AB_1089179 | BioLegend, San Diego, CA, USA |
| PCNA | PC10 | Mouse | RRID:AB_2160651 | Agilent, Santa Clara, CA, USA |
| PTEN |  | Rabbit | RRID:AB_86763 | Innovative Research, Novi, MI, USA |
|  |  |  |  |  |

*Antibody used for immunostaining of both human and murine cells.
