# Supplementary material for: CRISPR/Cas9-mediated deletion of Interleukin-30 suppresses IGF1 and CXCL5 and boosts SOCS3 reducing prostate cancer growth and mortality
Source: J Hematol Oncol. 2022 Oct 13;15:145. doi: 10.1186/s13045-022-01357-6 (PMC9559017; doi:10.1186/s13045-022-01357-6)
Supplement: Supplementary file 2 — Additional file 2. Supplemental Figure 1 (Neutralization of IGF1 by Human IGF-1 antibody) and 2 (A.B. Cytofluorimetric analyses of androgen receptor expression in human PC cells DU145 and PC3. C.D. Western blot analyses of STAT1 and STAT3 protein expression in IL30-DU145 and IL30-PC3 cells). [file 13045_2022_1357_MOESM2_ESM.docx]

Supplemental Figure S1


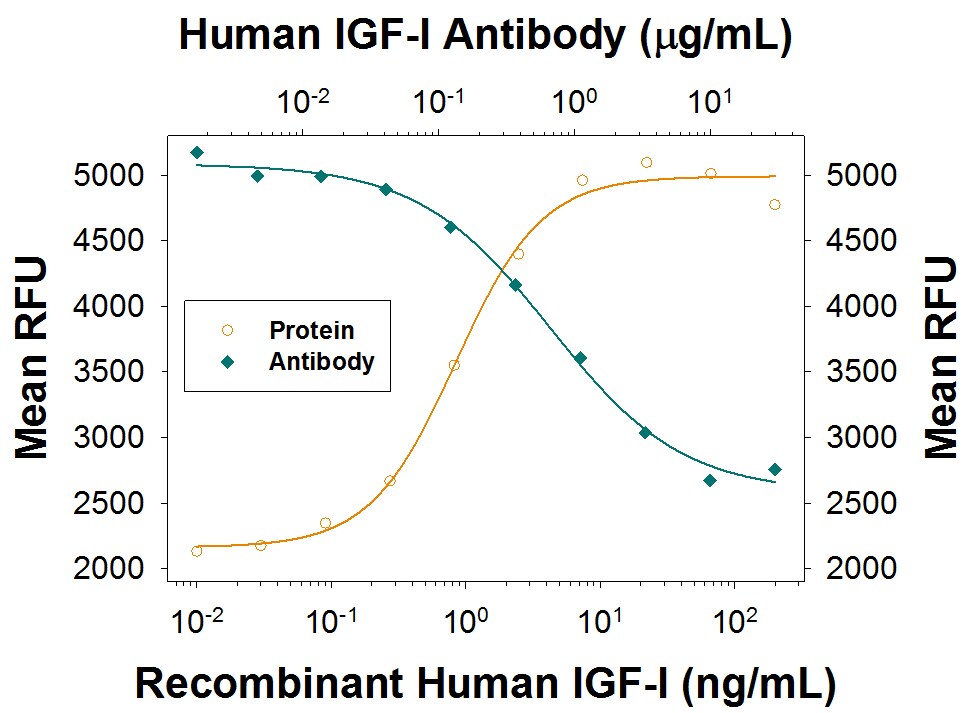


Neutralization of IGF1-induced proliferation in the MCF‑7 human breast cancer cell line by Human IGF-I/IGF-1 antibody (MAB2912, R&D Systems, Minneapolis, MN, USA). RFU: relative fluorescence units. From *https://www.rndsystems.com/products/human-igf-i-igf-1-antibody-997121_mab2912* (publicly available).


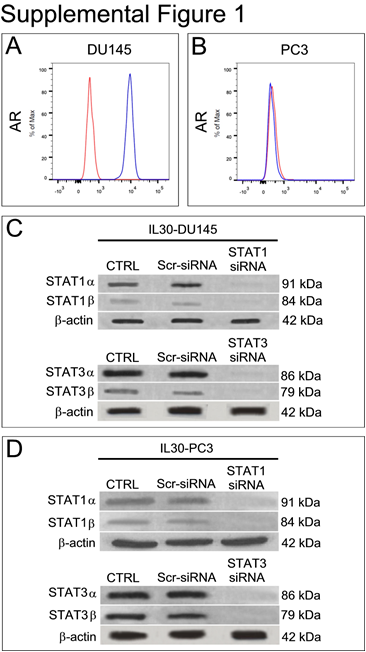
Supplemental Figure S2

**A. B.** Cytofluorimetric analyses of androgen receptor (AR) expression in human PC cells DU145 and PC3. (A) showing the expression of AR in DU145 and (B) its absence in PC3 cells. Red lines: isotype control. Blue lines: anti-AR Abs. Experiments were performed in triplicate.

**C. D.** Western blot analyses of STAT1 and STAT3 protein expression in IL30-DU145 (C) and IL30-PC3 (D) cells, after silencing of STATs with specific siRNA. CTRL: untreated IL30-DU145, or untreated IL30-PC3 cells. Scr-siRNA: scrambled siRNA.
